# New Amber Fossils Indicate That Larvae of Dermestidae Had Longer Defensive Structures in the Past

**DOI:** 10.3390/insects16070710

**Published:** 2025-07-10

**Authors:** Jéhan Le Cadre, Joshua Gauweiler, Joachim T. Haug, Sofía I. Arce, Viktor Baranov, Jörg U. Hammel, Carolin Haug, Uwe Kaulfuss, Christine Kiesmüller, Ryan C. McKellar, Patrick Müller, Marie K. Hörnig, Ana Zippel

**Affiliations:** 1 Biocenter, Ludwig-Maximilians-Universität München (LMU Munich), Grosshaderner Str. 2, 82152 Planegg-Martinsried, Germany; jehan.lecadre@palaeo-evo-devo.info (J.L.C.); jhaug@bio.lmu.de (J.T.H.); chaug@bio.lmu.de (C.H.); 2 Cytology and Evolutionary Biology, Zoological Institute and Museum, University of Greifswald, Soldmannstr. 23, 17489 Greifswald, Germany; 3 GeoBio-Center at LMU, Richard-Wagner-Str. 10, 80333 München, Germany; 4 Doñana Biological Station EBD-CSIC, 41092 Seville, Spain; 5 Institute of Materials Physics, Helmholtz-Zentrum Hereon, Max-Planck-Str. 1, 21502 Geesthacht, Germany; 6 Department of Animal Evolution and Biodiversity, University of Göttingen, Untere Karspüle 2, 37073 Göttingen, Germany; uwe.kaulfuss@uni-goettingen.de; 7 Faculty of Biology, Institute of Evolutionary Biology, University of Warsaw, ul. Żwirki i Wigury 101, 02-089 Warszawa, Poland; 8 Royal Saskatchewan Museum, 2445 Albert St., Regina, SK S4P 4W7, Canada; 9 Independent Researcher, Kreuzbergstr. 90, 66482 Zweibrücken, Germany; 10 Medical Biology and Electron Microscopy Center, University Medical Center Rostock, Strempelstr. 14, 18057 Rostock, Germany

**Keywords:** Amber, Skin beetles, Carpet beetles, Larder beetles, Morphological diversity, hastisetae, defensive setae

## Abstract

Representatives of Dermestidae (skin, larder, and carpet beetles) play a crucial role as decomposers in global ecosystems, facilitating the recycling of animal and plant biomass to sustain nutrient cycling. Despite their widespread ecological presence and functional importance, the fossil record of their larval stages has remained sparse, with previous documentation limited to occasional discoveries. This study significantly expands the larval fossil record by identifying 36 amber-preserved specimens from the Cretaceous, Eocene, and Miocene time slices, obtained from deposits distributed globally. By challenging the historical view of larval fossil rarity, we reveal morphological changes in defensive setae over geological time, demonstrating that Cretaceous and later fossil larvae possess significantly longer absolute and relative setal lengths compared to their extant counterparts. These findings, bolstered by quantitative comparisons of setal and body dimensions across fossil and extant representatives, indicate evolutionary adaptations in defensive structures dating back at least 100 million years. Our results offer new insights into the paleobiology of the group Dermestidae, highlighting how the morphology of larvae potentially reflects historical ecological pressures and resources availability. This study emphasizes the importance of integrating fossil evidence with comparative morphology to elucidate the evolutionary trajectories and functional roles of larvae in ancient terrestrial ecosystems.

## Simple Summary

This study explores the fossil larvae of Dermestidae (skin, larder, and carpet beetles), which are essential in the decomposition of organic materials and play a significant role in nutrient cycling. While these beetles are well-known for their ecological contributions today, fossilized larvae have been relatively understudied. This paper reports the discovery of 35 new amber fossil specimens, and an additional possible larva, of Dermestidae. Many specimens are preserved in 100-million-year-old amber, collected from various global deposits. Our findings challenge the long-held belief that such fossils are rare. In addition to documenting the new specimens, our study also delves into the defensive adaptations of these larvae, focusing on their specialized hairs called hastisetae. Comparisons are made between the fossilized and modern larvae, revealing that over time, the defensive setae of Dermestidae have changed toward shorter defensive hairs and larger bodies. These findings not only contribute to the understanding of the evolutionary history of these larvae but also provide valuable insights into their ecological roles in ancient environments. Through this expanded fossil record, the research sheds light on how these beetles and their larvae adapted over millions of years and their ongoing importance in ecological processes.

## 1. Introduction

Representatives of Dermestidae, also commonly known as skin, hide, khapra, larder, leather, or carpet beetles, are omnipresent and ubiquitous around the world [1]. These beetles are often regarded as pests, as some of them occur in stored products [2,3,4], silk production [5,6], and other materials (exhaustive list in [7]). Larvae of Dermestidae, similar to other larvae of holometabolan insects, differ significantly in their habitus from the rather typical beetle-like habitus of their adults. The larvae appear extremely “hairy” due to their highly specialized defense mechanism: they use a various array of modified setae, called spicisetae and hastisetae, to repel or entangle their predators [8]. This kind of defense is quite unique among beetle larvae but is rather similar to the mechanical defense of bristly millipedes (Polyxenida). The defensive setae give both the larvae of Dermestidae and the representatives of Polyxenida an overall “hairy” look that sometimes leads to a misidentification of the specimens, at least on first sight.

A “typical” hastiseta has an elongate proximal part, a pedicel, that can bear more than 30 rosettes of sharply pointed scales (imbricate and triangular in lateral view), and distally it bears a distinctive “head” [9,10]. The pedicel is hollow, forming an internal canal extending into the hastiseta head [11]. This hastiseta head has several proximal, oval-to-elliptical, knob-like processes with stalks extending distally toward the tip. These structures appear to be connected by cytoplasmic strands [10]. In some larvae, the “knobs” are less apparent, giving the hastiseta head a more concealed look, with inner structures concealed by petal-like parts. This peculiar mechanical defense has proven to be quite effective to protect the larva and deter predators such as mites, beetles [12], ants, or spiders, and it can even lead to the death of the predator [13]. The presence of hastisetae in human consumption has also been considered a potential health hazard (i.e., allergic reaction) [14,15,16]. The presence of representatives of Dermestidae in museums has been intensively investigated due to the important damage that they can cause to items in natural history collections and books [17,18,19]. These beetles feed on decaying organic matter, fungi, pollen, and predominantly on carcasses [20]. Some species live in soil, where they also play an important role in decomposing carcasses [21]. Therefore, the beetles are also used as a tool to clean living tissues off of bones [22,23]. Additionally, Dermestidae is an important and indicative group for entomological forensic studies [24], as the presence of representatives of different species at different developmental stages can be used as an indicator of the postmortem time interval [24,25].

The omnipresence of representatives of Dermestidae in the modern fauna should indicate that they were already quite widespread in the past. However, their fossil record has been considered sparse, especially for larvae [26,27]. The oldest adult representative was reported from the middle Jurassic of China [28], yet there have been some older records suggested from traces; see [26,29,30]. More adults of Dermestidae have been reported from ambers of different outcrops [31]. This includes Miocene Dominican amber [32,33], Eocene Baltic amber (table p. 56, [34]) [35,36,37,38], and Cretaceous Kachin Myanmar amber [39,40], but also other deposits with exceptional preservation, such as the Eocene Eckfeld Maar [41]. Besides adults, indirect indications of fossil larvae of Dermestidae are also available in the literature, mainly in the form of highly modified hastisetae [10,42,43]. In addition, possible borings of larvae of Dermestidae have been reported on bones from various time slices, spanning from historical times back to the Jurassic [44,45,46,47,48,49,50,51,52,53,54,55,56,57,58]. However, the identification of such traces from the larvae of Dermestidae in fossils is very difficult and has to follow a few prerequisites [59]. For example, the extant representatives of *Dermestes maculatus* DeGeer, 1774 do modify bones, but linking their traces to the fossil record can be challenging [59].

The direct fossil record of larvae of Dermestidae is much rarer than of adult body fossils. On average, 10–15 fossil adults are found per one fossil larva recovered [27,60]. Larvae are, in general, less often reported due to identification challenges, and often, they are only mentioned but not figured in the literature [61,62,63]. To date, only a few fossils of larvae closely related to extant larvae of Dermestidae have been identified [27,31], such as *Anthrenus larvalis* Cockerell, 1917 [64], *Trogoderma larvalis* Háva, Prokop et Herrmann, 2006 [35], or larva of *Trinodes* Dejean, 1821 [65], with the oldest larva recorded from the Cretaceous period (i.e., *Anthrenus larvalis*). However, all identified larvae are considered *incertae sedis.*

Here, we provide an overview of the known fossil records of skin, larder, and carpet beetle larvae. In addition, we report new specimens of these beetles (and possibly a representative of a related group) in amber from different localities, including the Dominican Republic, Germany, New Zealand, the Baltic coast, Canada, and Myanmar. We discuss these findings and the actual fossil record of larvae of Dermestidae in amber. Additionally, we discuss the intriguing defense by specialized setae of larvae of Dermestidae and highlight the morphological diversity of these setae and how they have changed from the Cretaceous period till today.

## 2. Materials and Methods

### 2.1. Material

For our analysis, we investigated a dataset of 59 specimens of fossil larvae of Dermestidae (Supplementary Table S1) and 11 extant specimens of larvae of Dermestidae from the literature [66,67,68,69,70,71] (Supplementary Table S1). Partial fossil material was previously published as fossil larvae of Dermestidae [43,72,73], but further identification was not provided. Therefore, we investigated three of the previously published larvae and refigured them. In addition, 36 completely new fossils from Miocene German (Lausitz), Miocene Dominican, Miocene New Zealand (Hyde, Roxburgh), Eocene Baltic, Cretaceous Canadian (Grassy Lake), and Cretaceous Myanmar (Kachin) ambers were investigated and statistically analyzed in the scope of this publication. Additionally, six fossilized larvae of Dermestidae, preserved in Eocene Baltic amber, are figured in this publication (Supplementary Figures S1 and S2). These specimens were available to us only as images. Due to the uncertain location of some of these specimens, these were only figured but not further studied nor analyzed.

**Figure 1:**
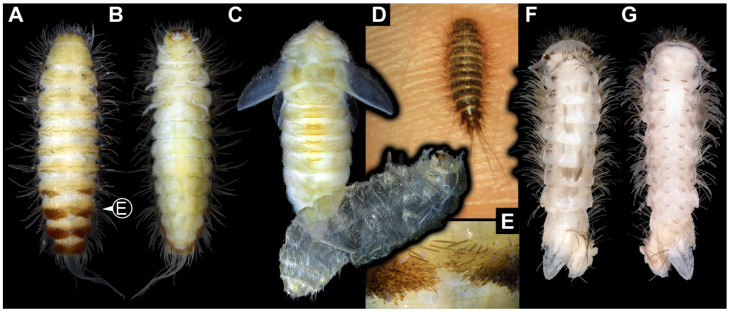
Extant immatures of Dermestidae and representatives of Polyxenida. (**A**–**C**,**E**) *Trogoderma versicolor* (ZMH 62859). (**A**) Habitus of larva in dorsal view. (**B**) Habitus of larva in ventral view. (**C**) Habitus of pupa in dorsal view, with larval exuvia still attached in ventro-lateral view. (**D**) Alive larva on the hand of one of the authors, in dorsal view. (**E**) Close-up of brushes of hastisetae and several spicisetae. (**F**,**G**) *Phryssonotus novaehollandiae* (Synxenidae). (**F**) Habitus of specimen in dorsal view. (**G**) Habitus of specimen in ventral view.

We also figured an extant adult of *Phryssonotus novaehollandiae* Silvestri, 1923 (Polyxenida) and extant representatives (larvae and a pupa with an exuvia) of Dermestidae (Figure 1) for a comparison with the fossil larvae. The single extant specimen of *P. novaehollandiae* preserved in alcohol was donated by Megan Short, Melbourne. The specimen is stored under the number ZSMA20250651, at the Zoologische Staatssammlung München (ZSM), Germany. Two of the extant specimens of Dermestidae (Figure 1A–C,E) are from the Zoological Collection of the Centrum für Naturkunde (CeNak), Leibniz-Institut zur Analyse des Biodiversitätswandels (LIB), formerly Zoological Museum Hamburg (ZMH 62859), Germany, and the other extant larva was captured by one of the coauthors (JTH), photographed and then released again. Two fossil specimens of Polyxenida were also figured. One fossil immature of Polyxenidae is preserved in Canadian amber (TMP 96.9.393) and one fossil adult of Synxenidae in Kachin Myanmar amber (PED 1112; Ludwig-Maximilians-Universität München).

Twenty-seven specimens in Dominican and Kachin Myanmar ambers are deposited in the Palaeo-Evo-Devo Research Group Collection of Arthropods, Ludwig-Maximilians-Universität München (PED), Germany. Four further specimens are part of private collections: three in the private collection of one of the coauthors (PM; BUB 3184, BUB 3346, BUB 3353) and one in the private collection of Christian Otto, Germany (Lausitz specimen); these specimens are available on request. Two specimens in Baltic ambers (SNSB BSPG 2018 III 40, SNSB BSPG 2018 III 142) are housed in the Staatliche Naturwissenschaftliche Sammlungen Bayerns, Bayerische Staatssammlung für Paläontologie und Geologie (SNSB BSPG), München, Germany. Two specimens from New Zealand amber (OU 33160.1, OU 33636.3) are deposited in the Geology Museum of the Geology Department, University of Otago (OU), New Zealand. Three specimens in Canadian amber (TMP 96.9.393a, TMP 96.9.393b, TMP 96.9.366) are housed in the Royal Tyrrel Museum of Palaeontology in Drumheller (TMP), Canada.

All PED ambers included in this publication were legally acquired on eBay from the seller burmite-miner. The images in the Supplementary Figures S1 and S2 were acquired from the owner of the website https://www.ambertreasure4u.com/ (accessed on 8 September 2024) and were processed with permission from the owner.

### 2.2. Methods

Fragile Canadian amber pieces were vacuum-embedded in a mineralogical-grade epoxy (Epo-Tek 301), ground with lapidary wheels to create optimal viewing angles, and then affixed to petrographic support slides with the same epoxy. The petrographic slides were imaged using a motorized camera lift with a Canon 5D Mark III digital camera (Canon Inc., Tokyo, Japan), using either a Canon MP-E 65 mm f/2.8 1–5× lens (Canon Inc., Tokyo, Japan) or a K2 long distance microscope (Infinity Photo-Optical Company, Centennial, CO, USA) bearing a Mitutoyo 10× or 20× Plan Apo compound microscope lens (Mitutoyo Corporation, Kawasaki, Japan). 

The remaining new specimens were documented using a Keyence VHX-6000 digital microscope (Keyence Corporation, Osaka, Japan) at different magnifications (×200–×500). We used multiple illumination settings (coaxial polarized light, ring light, transmitted light) with different backgrounds (black, white, glass). Polished amber pieces were covered by a drop of glycerol followed by a coverslip to reduce the light refraction from the curves of ambers (see methods in [74]). We additionally trimmed the amber piece PED 3663 because the fine details of the head were inaccessible due to the unfortunate partially embedded position of the specimen within the amber piece. Rough grinding and cutting were performed with an electric hand-held rotary tool (Ferm FCT-300 Combitool) (FERM International B.V., Zwolle, Netherlands). Further grinding was carried out using abrasive paper of different grits. Final polishing was performed with silver polish (Poliboy) (POLYBOY GmbH, Lilienthal, Germany). The stacked images were obtained using the built-in Stack and HDR software of the Keyence microscope [75] or Helicon Focus v. 6.0.18 software.

We processed one specimen (OU 33636.3) with synchrotron radiation-based X-ray computed microtomography (SRμCT) because it allows high-resolution imaging of morphological structures (e.g., mouthparts) not accessible with light microscopy. This was performed on the Imaging Beamline P05 [76,77,78] operated by the Helmholtz-Zentrum Hereon at the PETRA III storage ring (Deutsches Elektronen Synchrotron—DESY, Hamburg, Germany), using a photon energy of 18 keV and a sample-to-detector distance of 100 mm. Projections were recorded with a custom 50 MP CMOS imaging system (XIMEA CB500MG-CM) with an effective pixel size of 0.92 µm. For each tomographic scan, projections were recorded at equal intervals between 0 and π. Reconstruction was carried out by applying a transport of intensity phase retrieval approach and using the filtered back projection algorithm (FBP). Pipeline was executed in a custom reconstruction pipeline using MATLAB ver. 24.2.0.2863752 R2024b (Math-Works) and the Astra Toolbox ver. 2.2 [79,80,81]. Raw projections were binned two times for further processing, resulting in an effective isometric pixel size of the reconstructed volume (voxel) of 1.83 µm. Scanned volumes were reconstructed using Drishti ver. 2.6.6 [82]. To decrease the strain on computer memory, we converted all the stacks into 8-bit tiffs, downscaled all of the tiffs by 50%, and then subsequently cropped the empty space around the amber piece using Fiji “scale” and “crop” functions [83]. Volumes were rendered in Drishti ver. 2.6.6 [82]. The additional Volrens of the mouthparts (Supplementary Figure S3) were reconstructed again with Drishti and Amira ver. 2024.1. [84].

Images were processed and optimized using the software Photoshop CS2 version 9.0 (9.0 × 211). Measuring of the specimens was performed with the software ImageJ (ver. 1.54k) and again the software Photoshop CS2.

### 2.3. Statistical Analysis

We measured and investigated the body lengths of the new fossil larvae, fossil larvae from the literature, and extant larvae (Supplementary Tables S1 and S2). All analyses were performed in the R-environment (ver. 4.4.2) [85] using the software RStudio (ver. 2024.9.0.375) [86]. We investigated the body length, the longest available setae, the longest available hastisetae, the ratio of seta–body length, and the ratio of hastisetae–body length. We examined at the level of Dermestidae, investigating the morphological diversity of the whole group. We also examined the morphological diversity within the group Megatominae, the only ingroup within Dermestidae with enough fossil specimens for the investigation of morphological changes over time. Due to the sampling size limitations, within the ingroups of Megatominae, further analyses were not possible.

As some fossil larvae of Dermestidae were not fully preserved, we decided not to include fossils with a fragmented body (e.g., head missing; specimens 6, 13, 23, 24, 28, 39, 40 and 57) in the analysis to limit potential bias in the body lengths and ratios. Yet, we computed the complete dataset and figures for comparing the results of the two datasets (see Supplementary Table S3). In addition, we did not consider the developmental stages of the larvae, as the sampling size is insufficient for clear identification (see Section 4 for more explanations).

We computed violin plots, using the package ggplot2 (ver. 3.5.1) [87], which represent the density of measurements and their variation, depending on the age of the specimen (extant, Miocene, Eocene, Cretaceous). For the comparison of the measurements between time periods, we used the packages ggpubr (ver. 0.6.0) [88], car (ver 3.1.3) [89], and FSA (ver. 0.9.6) [90], allowing us to perform Kruskal tests followed by Dunn’s post hoc test (Supplementary Table S3). While using Dunn’s test, we used the Holm–Bonferroni method for multiple comparison correction. In addition, we computed the mean of all measurements, plotted them on the violin plots, and traced lines between these values. For further processing of the data, in complement to ggplot2, we used the package radiant (ver. 1.6.6) [91].

### 2.4. Description Style for New Specimens

In the Results section, we used a descriptive style to make the identification of structures in the new specimens clear to both specialists and non-specialists. Where relevant, we included terms commonly used by coleopterologists for larvae of Dermestidae in brackets. To avoid confusion, we refrain from calling all body units “segments” due to unresolved developmental details in certain trunk regions. The same applies for the leg elements.

## 3. Results

We are aware of the political situation in Myanmar and its possible influence on the trade of amber (Letter of the Society of Vertebrate Paleontology, SVP) [92]. However, the direct influence on the amber trade has been disputed [93,94], and the usefulness of the originally proposed moratorium on research on amber [92] has been drawn into question [95]. In the case of small and incomplete specimens of very low monetary value, such as the new specimens of Dermestidae in Myanmar amber in this publication, no official documents for export and import were received. Even though we agree with the SVP that problems considering amber pieces from conflict zones should be addressed [96], the procedures suggested by the SVP do not work for small pieces with low monetary value. Therefore, we cannot provide the documents suggested by the SVP due to common administrative procedures.

### 3.1. General Description of Body Organization of the Larvae

Body elongate to spindle-shaped, slightly flattened, or circular in diameter. Body differentiated into head and trunk. Head composed of six segments, mostly discernible by appendages or derivative structures of the segment: ocular segment and post-ocular segments 1–5. Ocular segment discernible by clypeus and derivative structure, labrum, anteriorly, mostly clypeus parted from labrum by a clypeo-labral suture (so-called “free” labrum), sometimes without the suture. Post-ocular segments 1 and 3–5 with appendages (antennae, mandibles, maxillae, labium); post-ocular segment 2 (intercalary segment) without appendages. Antennae with three elements each (antennomeres). Head with mouthparts directed ventrally (hypognathous), completely or slightly drawn out anteriorly. Mandibles broad and stout or wedge-like, sometimes with a single tooth or two teeth. Each maxilla with cardo, stipes, two endites (lacinia and galea), and maxillary palp. Lacinia sickle-shaped, galea with many hairs (setae). Maxillary palp with three or four elements (palpomeres). Labium with prementum, mentum, submentum, and a pair of labial palps with two elements each (palpomeres). Labium at the functionally anterior end with ligular sclerite (ligula).

Trunk differentiated into anterior part (thorax) and posterior part (abdomen). Thorax with three segments and abdomen with eight segments and trunk end (bearing the anal membrane), in some representatives trunk end with paired urogomphi and/or with a pygopod (extended anal membrane). Thorax segments each with a pair of locomotory appendages (legs). Each leg with five elements: coxa, trochanter, femur, tibia, and tarsungulum.

Body bears multiple setae. Some representatives bear specialized setae, spicisetae, and hastisetae. Spicisetae are with rosettes of imbricate scales throughout length. Hastisetae are with “arrowhead”-shaped heads and triangular (in lateral view) structures along the entire length of the setae.

### 3.2. Probable Fossil Larvae of Dermestidae from the Miocene

#### 3.2.1. Specimen 1 [26] (Their Figure 1A–C)

Kiselyova and McHugh [26] provided micrographs (all on p. 471) of a larva of Dermestidae preserved in Dominican amber. Images include a lateral view (figure 1A), dorsal view (figure 1B), and details of the hastisetae (figure 1C).

#### 3.2.2. Specimens 2 and 3 [33] (Their Figures 21 and 22)

Poinar and Háva [33] provided a micrograph of two larvae of Dermestidae preserved in Dominican amber. Both were interpreted as representatives of the group *Apsectus* LeConte, 1854 and are accessible in lateral view. The first specimen (Specimen 2) has the accession number C-7-7A; according to the provided scale, the larva is 0.88 mm long, excluding the setae. The second specimen (Specimen 3) has the accession number C-7-7B; according to the provided scale, the larva is 0.88 mm long, excluding the setae.

#### 3.2.3. Specimen 4 [31] (Their Figures 10 and 11)

Háva [31] provided two images of a larva of Dermestidae in Dominican amber, one as an overview of the whole amber piece with the larva and other inclusions, and a close-up image of the larva. The larva was interpreted as a representative of the group *Cryptorhopalum* Guérin-Méneville, 1838. According to the text, the larva is 5.2 mm long.

#### 3.2.4. Specimen 5 (Lausitz Specimen, Figure 2 and Figure 3)

**Figure 2:**
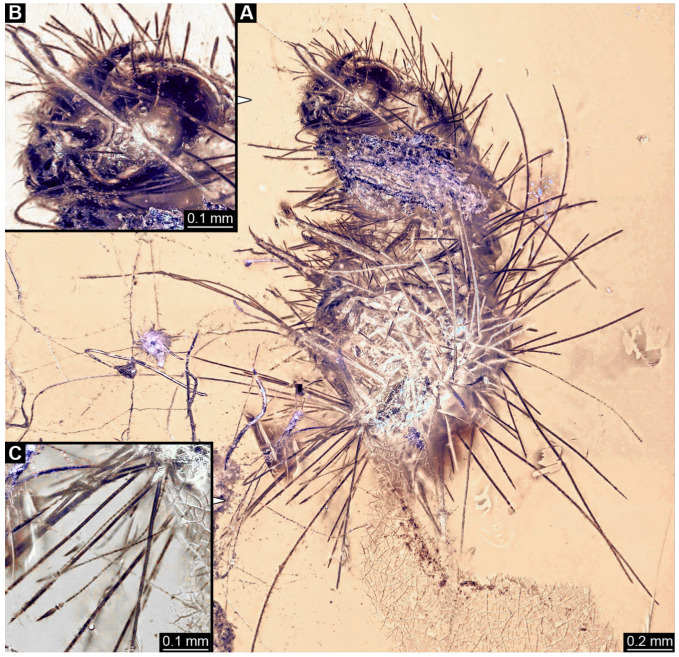
Specimen 5, fossil larva of Dermestidae in Miocene Lausitz German amber. (**A**) Habitus in ventral view. (**B**) Close-up of head with visible appendages and stemmata. (**C**) Close-up of hastisetae.

**Figure 3:**
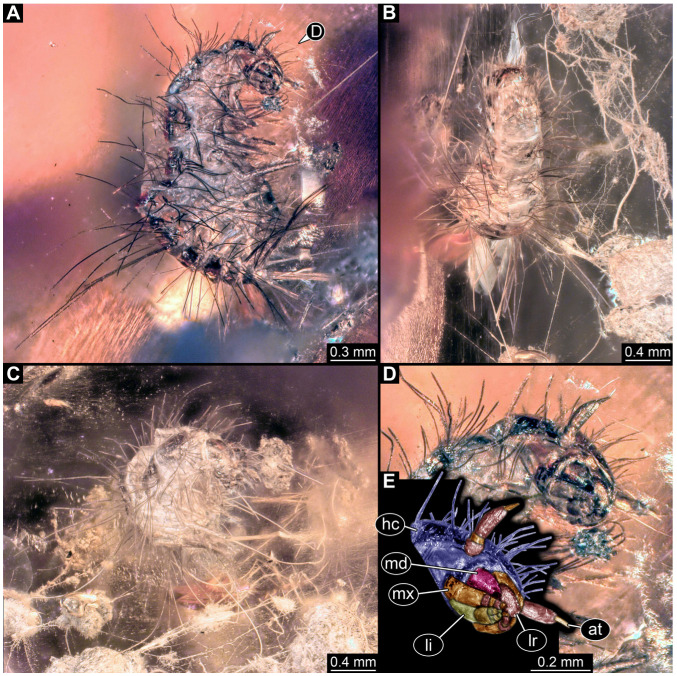
Specimen 5, fossil larva of Dermestidae in Miocene Lausitz German amber. (**A**) Habitus in ventro-lateral view. (**B**) Anterior part of the body in dorsal view. (**C**) Habitus in lateral view. (**D**) Close-up of head in lateral view. (**E**) Color-marked version of (**D**). Abbreviations: at = antenna; hc = head capsule; li = labium; lr = labrum; md = mandible; mx = maxilla.

A single specimen preserved in Lausitz amber, previously depicted in Zippel [72]. Total body length around 1.8 mm. Specimen appears completely preserved (Figure 2A and Figure 3A–C). Head with mouthparts directed ventrally partially discernible; possible stemmata discernible (Figure 2B). Both antennae discernible (Figure 3A,D,E), with three elements (antennomeres): ultimate element (antennomere 3) thinner than rest. Antenna in total shorter than head capsule. Mouthparts partially discernible: heavily sclerotized mandibles, partial maxillae maxillary palps with four elements each, labium with two discernible parts and palps with three elements (Figure 3D,E). Thorax segments each with a pair of locomotory appendages (legs; Figure 2A). Head and trunk bear long setae. Hastisetae discernible (Figure 2C). The longest seta is 1.1 mm long, the longest hastiseta is 0.3 mm long.

#### 3.2.5. Specimen 6 (PED 1589, Figure 4)

**Figure 4:**
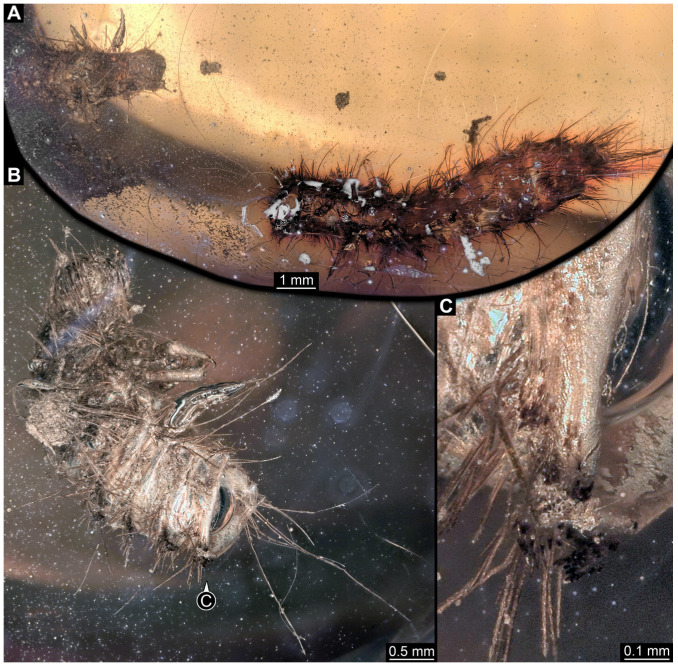
Specimens 6 and 7 (PED 1589), fossil larvae of Dermestidae in Miocene Dominican amber. (**A**) Habitus of two larvae in different views. (**B**) Habitus of specimen 6 in lateral view. (**C**) Close-up of hastisetae of specimen 6.

One of two specimens preserved in a single piece of Dominican amber (Figure 4A). Total body length around 4.2 mm. Specimen appears completely preserved but posterior trunk end not discernible (Figure 4A,B). Head probably with mouthparts directed ventrally, but no details discernible (Figure 4B). Thorax segments each with a pair of appendages (legs; Figure 4B). Head and trunk bear moderately long setae, several long setae posteriorly of trunk end (Figure 4B). Hastisetae discernible (Figure 4C). The longest seta is 3.9 mm long; the longest hastiseta is 1.4 mm long.

#### 3.2.6. Specimen 7 (PED 1589, Figure 4A and Figure 5)

**Figure 5:**
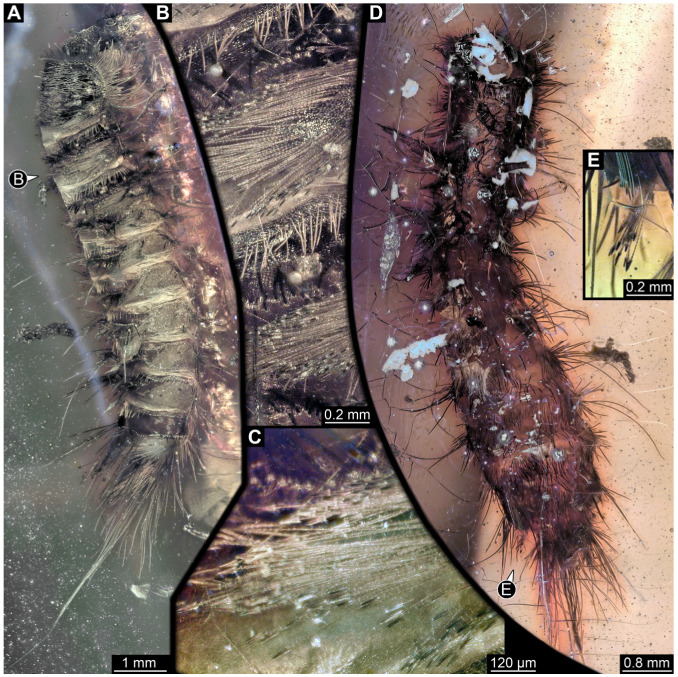
Specimen 7 (PED 1589), fossil larva of Dermestidae in Miocene Dominican amber. (**A**) Habitus in dorso-lateral view. (**B**) Rows of setae and hastisetae on thorax. (**C**) Close-up on hastisetae. (**D**) Habitus in ventral view. (**E**) Close-up on brush of hastisetae on abdomen.

One of two specimens preserved in a single piece of Dominican amber (Figure 4A). Total body length around 8.4 mm. Specimen appears completely preserved (Figure 4A and Figure 5A,D). No details discernible on head (Figure 5D). Thorax segments each with a pair of appendages (legs; Figure 5D). Head and trunk bear moderately long setae. Hastisetae discernible (Figure 5B,C,E). The longest seta is 5 mm long; the longest hastiseta is 1.4 mm long.

#### 3.2.7. Specimen 8 (OU 33160.1, Figure 6) (Their Figure 3I) [43]

**Figure 6:**
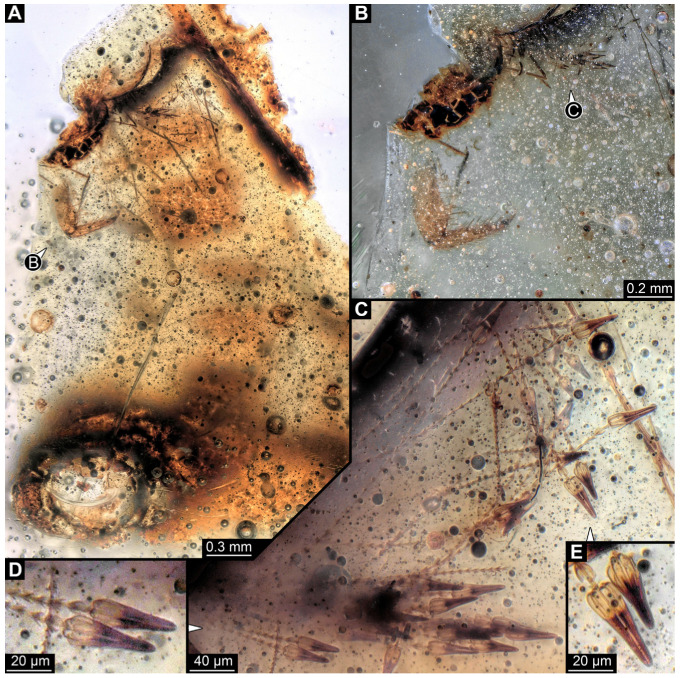
Specimen 8 (OU33160.1), fossil larva of Dermestidae in Miocene Roxburgh New Zealand amber. (**A**) Habitus, specimen incomplete, mostly preserving one trunk appendage (leg) and hastisetae (both top) and series of sternites of abdomen segments, in ventral view. (**B**) Close-up of a leg, fragmented mouthparts and hastisetae. (**C**) Close-up on hastisetae. (**D**,**E**) Close-ups of heads of hastisetae in lateral view.

Single specimen preserved in New Zealand amber previously published in Schmidt et al. [43]. Body incomplete; total body length not measurable. Specimen only fragmentarily preserved, damaged and partly disarticulated, likely representing an exuvia (Figure 6A). Partial head with discernible mouthparts directed ventrally (Figure 6A). No antennae discernible. Mouthparts partially discernible: heavily sclerotized, fragmented mandibles; partial maxilla, with maxillary palp (Figure 6B). Only a single fragmented locomotory appendage discernible (a leg; Figure 6A,B). Head bears moderately short setae. Hastisetae discernible (Figure 6C–E). The average length of accessible hastisetae is 0.25 mm.

#### 3.2.8. Specimen 9 (OU 33636.3, Figure 7 and Figure 8)

**Figure 7:**
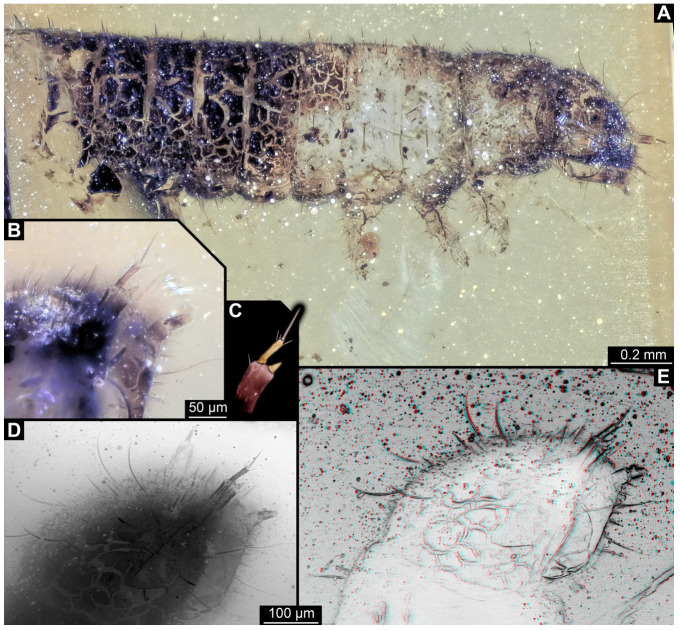
Specimen 9 (OU33636.3), fossil larva of Dermestidae in Miocene Hyde New Zealand amber, posterior part of the body torn apart and not available. (**A**) Partial habitus in lateral view. (**B**) Close-up of head with antenna and maxillary palp. (**C**) Color-marked antenna. (**D**) Grayscale image of head in dorso-lateral view. (**E**) Red-cyan stereo anaglyph of head in dorso-lateral view (use red–cyan glasses for the 3D effect).

**Figure 8:**
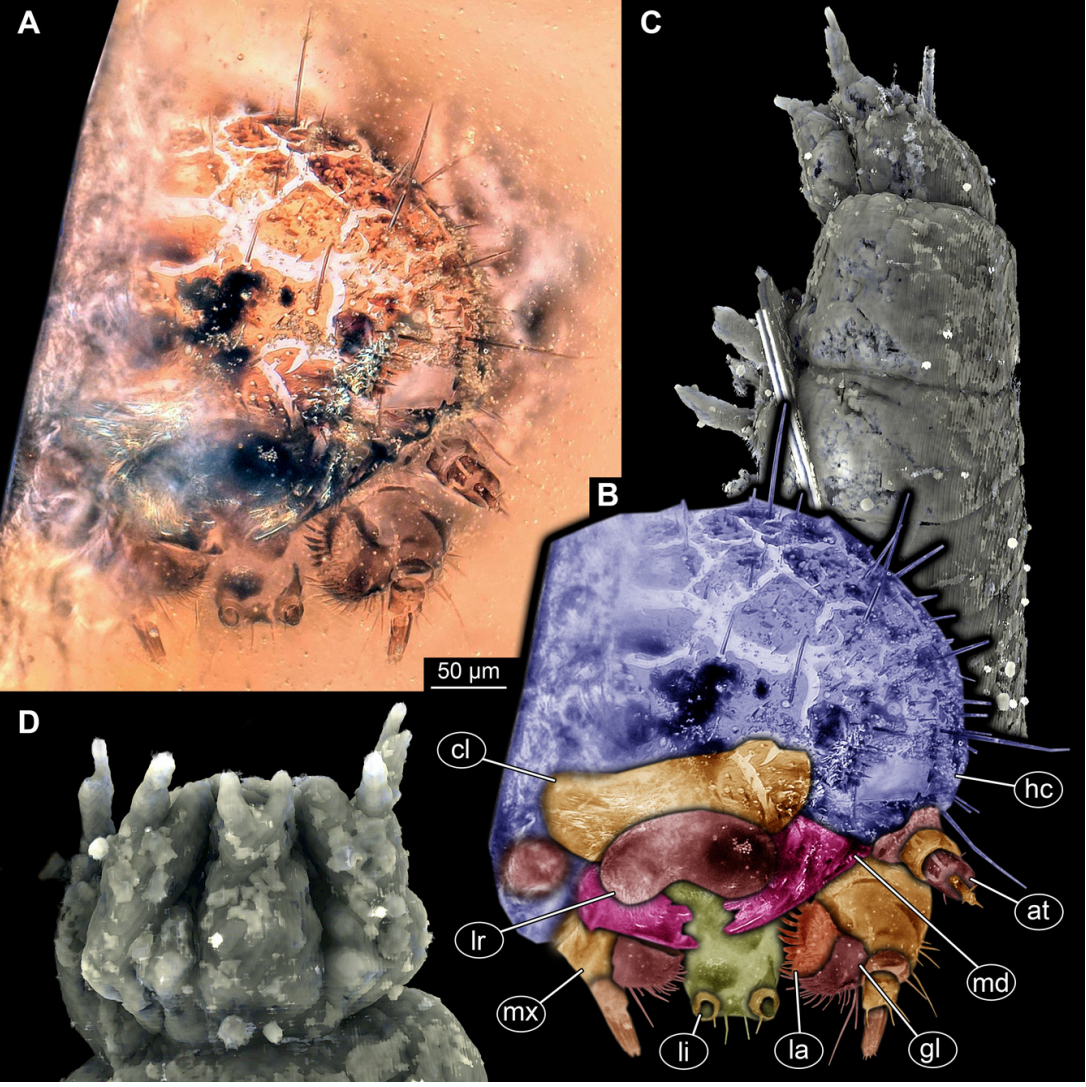
Specimen 9 (OU33636.3), fossil larva of Dermestidae in Miocene Hyde New Zealand amber. (**A**) Close-up of head, in anterior view. (**B**) Color-marked version of (**A**). (**C**) Volume rendering of habitus in lateral view based on synchrotron X-ray tomography. (**D**) Volume rendering of head in ventral view. Abbreviations: at = antenna; cl = clypeus; gl = galea; hc = head capsule; la = lacinia; li = labium; lr = labrum; md = mandible; mx = maxilla.

Single specimen preserved in New Zealand amber; body incomplete. Length of preserved body part around 2 mm. Specimen fragmentarily preserved, with only head and first nine trunk segments preserved (Figure 7A). Head probably with mouthparts directed ventrally (Figure 7A,D,E). Both antennae discernible (Figure 7B–E and Figure 8A–C), each with three elements (Figure 8A–C), with large cone-shaped sensory process on penultimate element (Figure 7B,C), and with ultimate element thinner than rest. Antenna, in total, shorter than head capsule. Clypeus and labrum discernible, parted by suture (Figure 8A,B). Mouthparts partially discernible: heavily sclerotized mandibles with two teeth each; partial maxilla, with endites (lacinia and galea; Figure 8B, Supplementary Figure S3) with setae and spines, and maxillary palp with at least three elements (palpomeres; Figure 8A,B); partial labium with palps with two elements (palpomeres; Figure 8A,B,D). Thorax segments each with a pair of locomotory appendages (legs; Figure 7A). Head and trunk bear short setae. Hastisetae not discernible. The longest seta is 0.1 mm long.

### 3.3. Fossil Larvae of Dermestidae from the Eocene

#### 3.3.1. Specimen 10 [35] (Their Figure 11)

Háva et al. [35] provided images of a larva of Dermestidae preserved in Baltic amber. The specimen is the holotype of the species *Trogoderma larvalis*. Images included a micrograph in dorsal view (figure 11, p. 283) and drawings of a hastiseta (figure 12, p. 285) and a distal part of a leg (figure 13, p. 285). The specimen has the accession number GPIH (type no. 4466); according to text, the larva is 3.8 mm long (p. 286; unclear if including setae). The measured longest seta is 0.5 mm long; hastisetae are discernible and estimated to be 1.2 mm in length.

#### 3.3.2. Specimen 11 [65] (Their Figures 1–4)

Kadej and Háva [65] provided micrographs of a larva of Dermestidae preserved in Baltic amber. The specimen was interpreted as a representative of the group *Trinodes*. Images include different dorsal views (figures 1 and 2, p. 3) and ventral views (figures 3 and 4, p. 4). The specimen has the accession number 5369; according to the text (p. 3), the larva is 1 mm long (unclear if including setae). The average length of setae is around 0.4 mm.

#### 3.3.3. Specimen 12 [97] (Their Figure 3)

Háva [97] provided an image of a larva of Dermestidae preserved in Baltic amber in dorso-lateral view (figure 3). The specimen was interpreted as representative of *Anthrenus* sp. No indication of size was provided. In the text (p. 220), it was stated that three specimens were examined, but it is unclear which of them is depicted in figure 3.

#### 3.3.4. Specimen 13 [98] (Their Figures 8 and 14F)

Grimaldi et al. [98] provided images of a larva of Dermestidae preserved in Alaskan amber (figures 8 and 14F). The specimen was interpreted as a representative of the group Megatominae. Images include an overview of the incomplete specimen, micrographs of plumose setae and hastisetae (figure 8), and a drawing of one hastiseta (figure 14F). Head and legs are missing. The specimen has the accession number AMNH LC-II_B4.

#### 3.3.5. Specimen 14 [60] (Their Figures 1 and 2)

Perkovsky et al. [60] provided images of an exuvia of a larva of Dermestidae preserved in Sakhalinian amber. The specimen is the holotype of the species *Trogoderma ainu* Perkovsky, Háva et Zaitsev, 2021. Images include different dorsal views (figures 1 and 2), details of head (figures 3 and 4), antenna (figure 5), hastisetae (figure 6), ventral sclerites (figure 7), and foreleg (figure 8). According to the text (p. 189) and figure 1, the larva is 1 mm long (excluding setae). The specimen has the accession number 3387/1060. The measured length of the longest seta is around 0.9 mm, and 0.2 mm for the hastiseta.

#### 3.3.6. Specimen 15 (SNSB BSPG 2018 III 40, Figure 9A)

**Figure 9:**
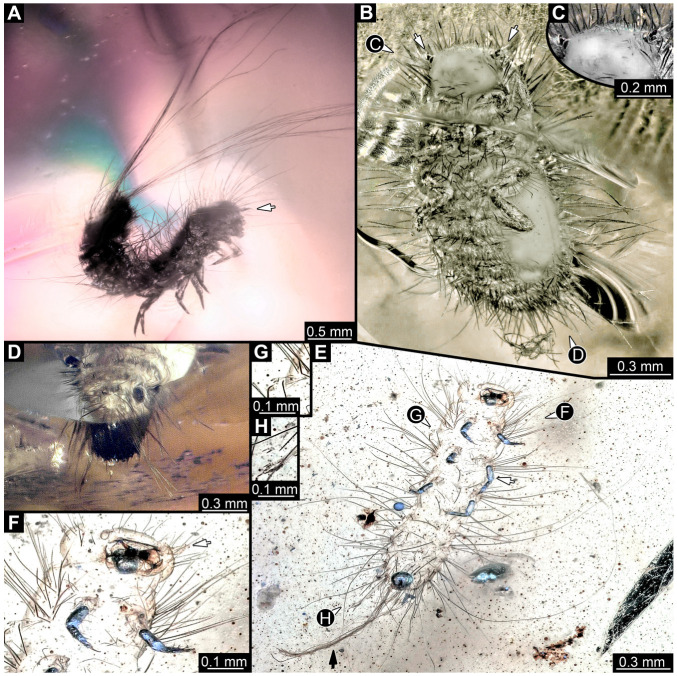
Fossil larvae of Dermestidae. (**A**–**D**) Eocene Baltic amber. (**E**–**H**) Cretaceous Kachin Myanmar amber. (**A**) Specimen 15 (SNSB_BSPG 2018 III 40), habitus in lateral view, head with antenna (arrow). (**B**–**D**) Specimen 16 (SNSB BSPG 2018 III 142). (**B**) Habitus in ventral view with head capsule with antennae (arrows). (**C**) Close-up of the head. (**D**) Brush of specialized setae at the posterior end of abdomen in posterior view. (**E**–**H**) Specimen 49 (BUB 3346). (**E**) Habitus in ventral view with legs (white arrow) and posterior brush of setae (black arrow). (**F**) Close-up of antenna (arrow) and mouthparts; (**G**) Close-up of hastisetae on prothorax. (**H**) Close-up of hastisetae on abdomen.

Single specimen preserved in Baltic amber. Total body length around 2.5 mm. Specimen completely preserved (Figure 9A). Head probably with mouthparts directed ventrally. One antenna discernible (Figure 9A: white arrow), with three elements (antennomeres). Antenna in total shorter than head capsule. Thorax segments each with a pair of locomotory appendages (legs; Figure 9A). Head and trunk bear sharp, moderately long setae. Posterior end of trunk with a possible broad and dense tuft of hastisetae, and brush of long setae discernible posterior to the trunk end (Figure 9A). The longest seta is 3.2 mm long.

#### 3.3.7. Specimen 16 (SNSB BSPG 2018 III 142, Figure 9B–D)

Single specimen preserved in Baltic amber. Total body length around 1.35 mm. Specimen completely preserved but partially covered by Verlumung (cloudy amber, Figure 9B). Head probably with mouthparts directed ventrally. Both antennae discernible (Figure 9B: arrows, Figure 9C). Antenna in total shorter than head capsule. Thorax segments each with a pair of locomotory appendages (legs; Figure 9B). Head and trunk bear moderately long setae and spicisetae (Figure 9B). Posterior end of trunk with a broad and dense tuft of hastisetae (Figure 9D). The longest seta is 0.5 mm long; the longest hastiseta is 0.2 mm long.

Additional specimens of fossil larvae in Baltic amber are available from the amber trader Marius Veta on his website https://www.ambertreasure4u.com/ (accessed 8 September 2024). The website provides multiple micrographs of entire larvae of Dermestidae (these six specimens are identified on the website as five representatives of Megatominae and one representative of Trinodinae) in ventral and dorsal views (Supplementary Figures S1 and S2). Additionally, some morphological characters of specimens are available as close-up micrographs. We did not study these larvae further nor use them in the analyses (for explanation see Section 2.1 of the publication); therefore, we attributed no specimen numbers to these fossils. Nevertheless, these specimens additionally show the morphological diversity of larvae of Dermestidae in the Eocene and how common they are.

### 3.4. Fossil Larvae of Dermestidae from the Cretaceous

#### 3.4.1. Specimen 17 [10] (Their Figure 1A,B)

Poinar and Poinar [10] provided micrographs of isolated hastisetae in Kachin Myanmar amber. Images include micrographs of total hastisetae (figure 1A,B), and details of shaft (figure 1C) and head (figure 2) of hastisetae. The same specimen was re-figured in Rasnitsyn et al. [99].

#### 3.4.2. Specimen 18 [42] (Their Figure 6A–D)

Peñalver et al. [42] provided images of isolated hastisetae as syn-inclusion to ticks (*Deinocroton draculi* Peñalver, Arillo, Anderson et Pérez-de la Fuente, 2017) in Kachin Myanmar amber (figure 6A–D). The isolated hastisetae are interpreted as representatives of Megatominae. The specimen has the accession number AMNH Bu-SA5.

#### 3.4.3. Specimen 19 [100] (p. 441)

Zhang [100] figured a larva of Dermestidae in Kachin Myanmar amber. No indication of size was provided.

#### 3.4.4. Specimen 20 [101] (Their Figure 29)

Poinar (figure 15) [102] figured a larva of Dermestidae adjacent to bird remains in amber. The image depicts the larva in dorsal view. No indication of size was provided. This specimen was re-figured by Poinar (figure 29) [101], with new information available: the beetle larva is preserved in Kachin Myanmar amber and, based on the scale available, is about 2.4 mm long, with the longest seta being about 1.2 mm.

#### 3.4.5. Specimen 21 [103] (Their Figure 4B)

Peris and Rust [103] provided a micrograph of a larva of Dermestidae in Kachin Myanmar amber. The specimen is depicted in dorso-lateral view (figure 4B). Based on the scale bar in figure 4B, the larva is about 1.8 mm long (excluding setae).

#### 3.4.6. Specimen 22 [104] (Their Figure 1)

Cockerell [64] described a larva of Dermestidae preserved in Kachin Myanmar amber, *Dermestes larvalis*. The specimen was first figured by Ross and York [105] and re-figured and re-described by Háva [104] as *Anthrenus larvalis*. The specimen has the accession number In. 19107-16. The image provided by Háva [104] is in ventral view; hastisetae are preserved, but no details are depicted. No indication of size was provided.

#### 3.4.7. Specimen 23 [31] (Their Figure 9)

Háva [31] provided a micrograph of a larva of Dermestidae in Kachin Myanmar amber. The specimen is depicted as an undescribed larva of *Trogoderma*. The larva is about 2.0 mm long.

#### 3.4.8. Specimens 24, 25, 26, 27 [106] (Their Figures 1C,D, 3 and S2)

Peñalver et al. [106] provided micrographs of exuvial remains of at least four larvae of Dermestidae in Spanish amber. Two were interpreted as remains of early instars of representatives of *Orphilus* Erichson, 1846. Specimen 24 (SJNB2012-31-01, figure 1C,D) is depicted in ventral view, and fragmentary preserved. Specimen 25 (SJNB2012-11, figure 3) is depicted in ventral (figure 3A,C,E) and lateral (figure 3B,D) view; according to the text, the larva is 0.83 mm long. Hastisetae are absent. Specimen 26 is an exuvium from El Soplao (ES-07-39). Specimen 27 is an exuvium from Peñacerrada I (MCNA 12063).

#### 3.4.9. Specimen 28 (TMP 96.9.366, Figure 10A,B)

**Figure 10:**
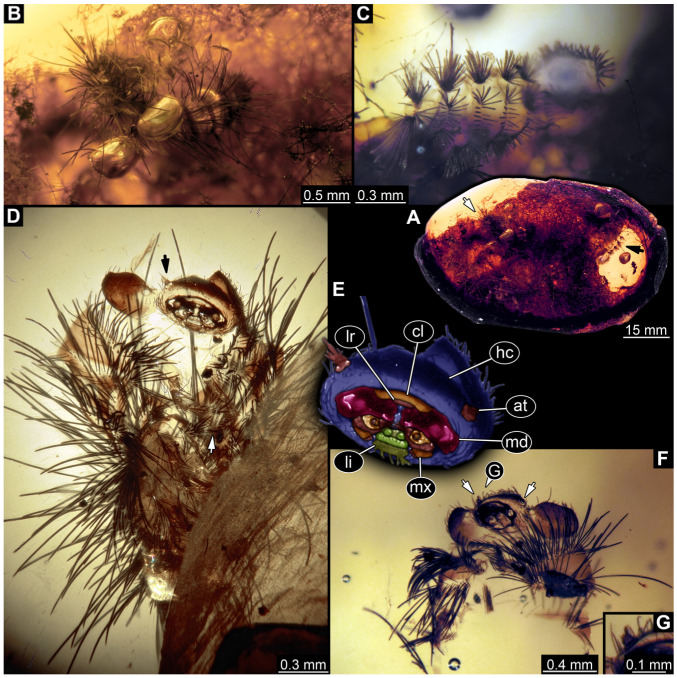
Representatives of Dermestidae (Coleoptera) and Polyxenidae (Polyxenida) in Canadian amber. (**A**) Single amber piece (TMP 96.9.366) with both a larva of Dermestidae (white arrow) and a probable larva of Polyxenidae (black arrow). (**B**) Close-up on the larva of Dermestidae (specimen 28) in dorso-lateral view from (**A**). (**C**) Close-up on the representative of Polyxenidae in dorsal view. (**D**) Specimen 29, an exuvia of a larva of Dermestidae (TMP 96.9.393a) in ventral view, head with antennae (black arrow) and legs with tarsungulum (white arrow). (**E**) Close-up and color-marked version of head capsule from (**D**). (**F**) Specimen 30, a single larva of Dermestidae (TMP 96.9.393b), torn apart, head with antennae (white arrows) and part of anterior trunk available in ventral view. (**G**) Close-up of antenna from (**F**). Abbreviations: at = antenna; cl = clypeus; hc = head capsule; li = labium; lr = labrum; md = mandible; mx = maxilla.

Single larval specimen of Dermestidae preserved in Canadian amber TMP 96.9.366 (Figure 10A). Amber contains densely organized organic material, presumably a cocoon, a larva of Dermestidae (Figure 10A: white arrow, Figure 10B), and an immature of Polyxenidae (Figure 10A: black arrow, Figure 10C) and the partial remains of an unidentifiable arachnid. Length of discernible part of body around 2 mm. Specimen completely preserved (Figure 10A,B) but only accessible in dorso-lateral view; head not accessible. Thorax segments each with a pair of locomotory appendages (legs; Figure 10B). Head and trunk bear long sharp setae and spicisetae. Hastisetae not discernible. The longest seta is 1.25 mm long.

#### 3.4.10. Specimen 29 (TMP 96.9.393a, Figure 10D,E)

One of two specimens preserved in Canadian amber piece TMP 96.9.393 (Figure 10D). Total body length not measurable. Specimen only fragmentarily preserved, damaged and partly disarticulated, likely representing an exuvia (Figure 10D). Head with mouthparts directed ventrally. Both antennae discernible (Figure 10D: black arrow, Figure 10E), each with three elements and large cone-shaped sensory process on penultimate element (antennomere 2); ultimate element (antennomere 3) thinner than rest. Antenna in total shorter than head capsule. Clypeus and labrum parted by suture. Mouthparts partially discernible: heavily sclerotized mandibles; maxilla with palps with several partial elements (palpomeres); labium with small palps (Figure 10D,E). Thorax segments each with a pair of locomotory appendages (legs; Figure 10D; white arrow). Head bears short setae and trunk bears long sharp setae. Spicisetae not discernible. Hastisetae not discernible. The longest seta is 1.2 mm long.

#### 3.4.11. Specimen 30 (TMP 96.9.393b, Figure 10F,G)

One of two specimens preserved in Canadian amber piece TMP 96.9.393. Total body length not measurable. Specimen only fragmentarily preserved, damaged and partly disarticulated, likely representing an exuvia (Figure 10F). Head with mouthparts directed ventrally. Both antennae discernible (Figure 10F: arrows, Figure 10G), with three elements (antennomeres) each (Figure 10G). Ultimate element (antennomere 3) thinner than rest. Antenna in total shorter than head capsule. Mouthparts partially discernible: heavily sclerotized mandibles; partial maxilla; partial labium (Figure 10F). Pro- and mesothorax fragmentarily preserved. Prothorax with a pair of locomotory appendages (legs; Figure 10F). Head bears short setae and trunk bears longer sharp setae in ring formation. Hastisetae not discernible.

#### 3.4.12. Specimen 31 (PED 2550, Figure 11A–D)

**Figure 11:**
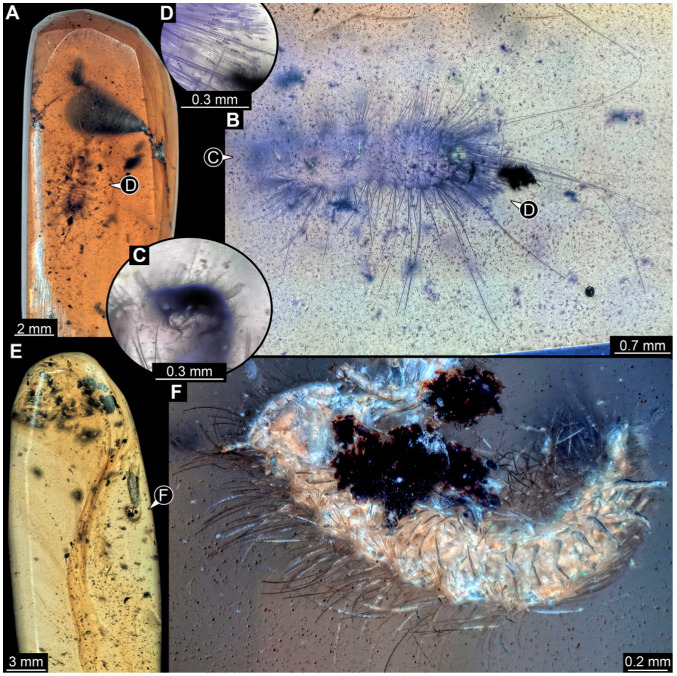
Representatives of Dermestidae (Coleoptera) and Synxenidae (Polyxenida) in Cretaceous Kachin Myanmar amber. (**A**–**D**) Larva of Dermestidae. (**A**) Amber piece with specimen 31 (PED 2550), larva of Dermestidae, and other syn-inclusions. (**B**) Habitus in ventral view. (**C**) Close-up of head with antenna. (**D**) Close-up of hastisetae. (**E**,**F**) Adult of *Phryssonotus* (Synxenidae). (**E**) Amber piece (PED 1112) with a bioinclusion of Synxenidae. (**F**) Habitus in ventro-lateral view.

Single specimen preserved in Kachin Myanmar amber. Total body length around 2.8 mm. Specimen is completely preserved but partially inaccessible and covered by other unidentified syn-inclusions (Figure 11A,B). Head (Figure 11C) probably with mouthparts directed ventrally. Both antennae discernible, with three elements (antennomeres) each (Figure 11C). Mouthparts not discernible. Thorax segments each with a pair of locomotory appendages (legs; Figure 11B). Trunk and likely head bear sharp setae and spicisetae; posterior segments of abdomen bear some extremely long setae, with lengths of up to 3.8 mm. Posterior end of trunk with a broad and dense tuft of hastisetae (Figure 11D). The longest hastiseta is 0.7 mm long.

#### 3.4.13. Specimen 32 (PED 1369, Figure 12)

**Figure 12:**
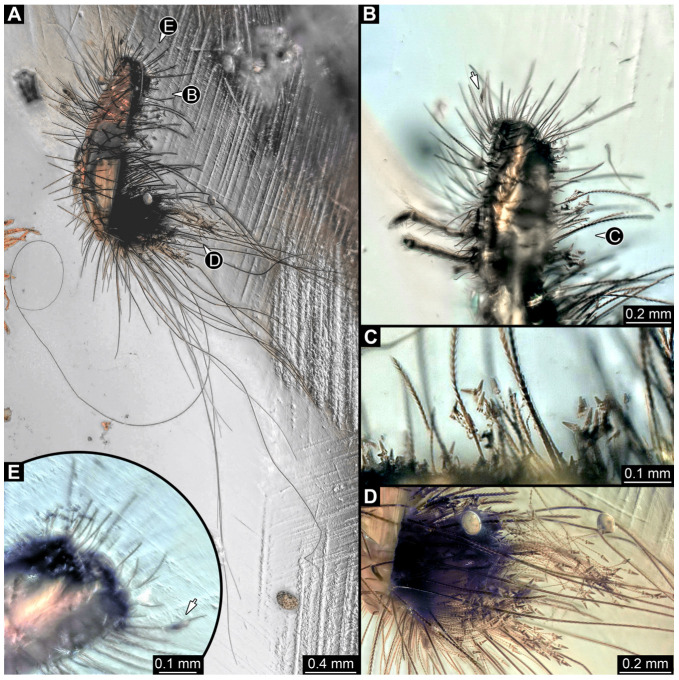
Specimen 32 (PED 1369), fossil larva of Dermestidae in Cretaceous Kachin Myanmar amber. (**A**) Habitus in lateral view. (**B**) Close-up of the head with antenna (arrow) and thorax with legs. (**C**) Close-up of spicisetae and hastisetae of thorax. (**D**) Close-up of posterior brush of hastisetae with surrounding spicisetae of abdomen. (**E**) Close-up of head with antenna (arrow) in ventral view.

Single specimen preserved in Kachin Myanmar amber, previously published in Haug et al. [73]. Total body length around 1.7 mm. Specimen complete (Figure 12A). Head probably with mouthparts directed ventrally. One antenna discernible (Figure 12B,E: arrows), possibly detached from the head capsule. Mouthparts not discernible. Thorax segments each with a pair of locomotory appendages (legs; Figure 12B). Head and trunk bear long, sharp setae, and spicisetae (Figure 12A,C). Trunk end has a broad and dense tuft of hastisetae posteriorly (Figure 12C,D). The longest seta is 3.4 mm long; the longest hastiseta is 0.7 mm long.

#### 3.4.14. Specimen 33 (PED 3504, Figure 13)

**Figure 13:**
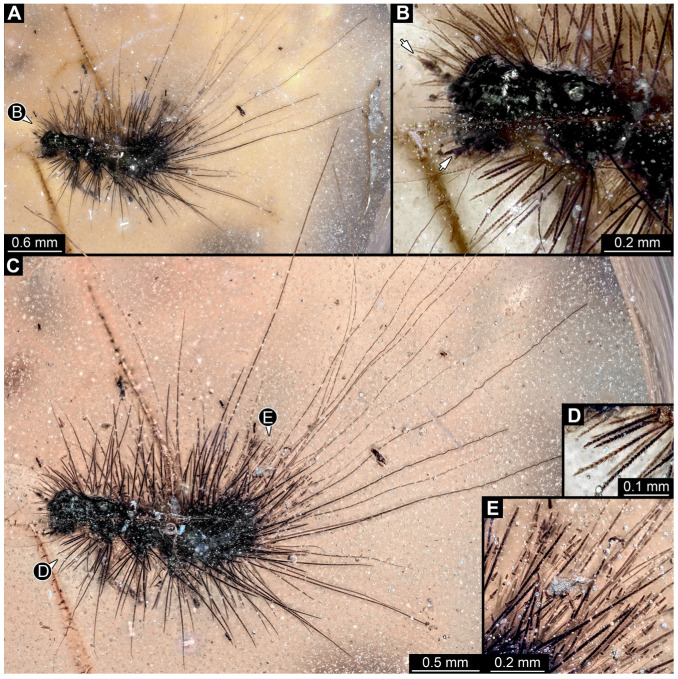
Specimen 33 (PED 3504), fossil larva in Cretaceous Kachin Myanmar amber. (**A**) Habitus in lateral view. (**B**) Close-up on the head with antennae (arrows) and prothorax. (**C**) Habitus photographed with higher magnification under ring light on the white background. (**D**) Close-up on spicisetae. (**E**) Close-up on hastisetae.

Single specimen preserved in Kachin Myanmar amber. Total body length around 1.55 mm. Specimen complete but partially covered by syn-inclusions (Figure 13A,C). Head (Figure 13B) probably with mouthparts directed ventrally. Both antennae discernible (Figure 13B: white arrow), with three elements (antennomeres) each. Antenna in total shorter than head capsule. Thorax segments each with a pair of locomotory appendages (legs; Figure 13A,C). Head and trunk bear long setae and spicisetae (Figure 13D). Hastisetae discernible (Figure 13E). The longest seta is 3 mm long; the longest hastiseta is 0.6 mm long.

#### 3.4.15. Specimen 34 (PED 3393, Figure 14A–H)

**Figure 14:**
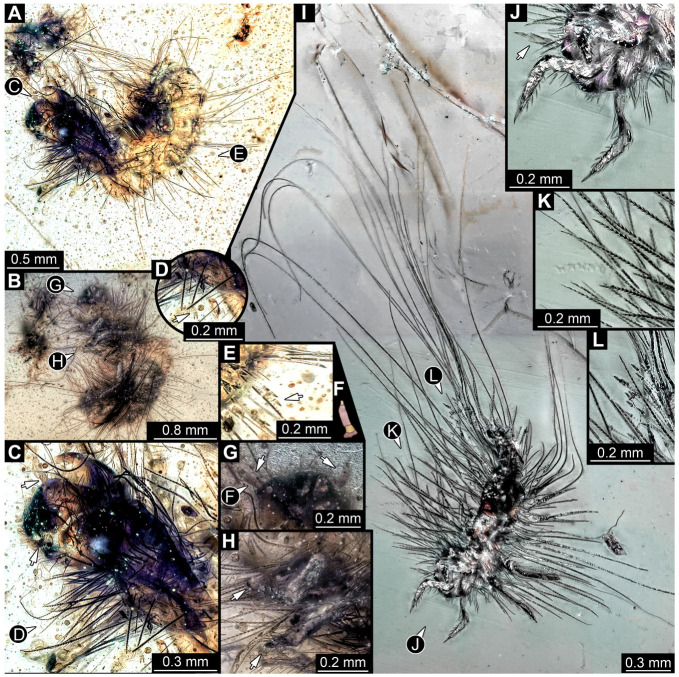
Fossil larvae in Cretaceous Kachin Myanmar amber. (**A**–**H**) Specimen 34 (PED 3393). (**A**) Habitus in dorsal view. (**B**) Habitus in ventral view. (**C**) Close-up of head capsule with both antennae (arrows) and prothorax in dorsal view, head capsule seems torn apart at moulting suture. (**D**) Close-up of hasti- and spicisetae of thorax, hastisetae with arrow-like heads (arrow). (**E**) Close-up of hasti- and spicisetae of abdomen, hastisetae with arrow-like heads (arrow). (**F**) Color-marked antenna from (**G**). (**G**) Close-up of head with antennae (arrows) in ventral view. (**H**) Close-up of legs with tarsungulum (arrows). (**I**–**L**) Specimen 35 (PED 2929). (**I**) Habitus in ventral view. (**J**) Close-up of the head with antenna (arrow) and first pair of legs in ventral view. (**K**) Close-up of the spicisetae. (**L**) Close-up of the hastisetae.

Single specimen preserved in Kachin Myanmar amber. Total body length around 2.4 mm. Specimen fragmentarily preserved, but body differentiated into head and trunk (Figure 14A,B). Head with mouthparts directed ventrally. Head capsule torn at the molting suture (Figure 14C). Both antennae discernible (Figure 14C,G: arrows), with three elements (antennomeres) each (Figure 14F). Antenna in total shorter than the head capsule. Mouthparts not discernible. Thorax segments each with a pair of locomotory appendages (legs; Figure 14B,H: arrows). Trunk bears sharp setae and spicisetae (Figure 14A,D); trunk end bears a tuft of long setae posteriorly (Figure 14A). Hastisetae discernible (Figure 14D,E: arrows). The length of the longest setae is not accessible due to entanglement on another unidentified inclusion; the longest hastiseta is 0.3 mm long.

#### 3.4.16. Specimen 35 (PED 2929, Figure 14I–L)

Single specimen preserved in Kachin Myanmar amber. Total body length around 1.3 mm. Specimen completely preserved (Figure 14I) but partially covered by an artifact. Head capsule not accessible, except for one antenna with three visible elements (antennomeres; Figure 14J: arrow). Thorax segments each with a pair of locomotory appendages (legs; Figure 14I). Head and trunk bear sharp setae and spicisetae (Figure 14K); posterior segments of abdomen bear some very long setae (Figure 14I). Hastisetae discernible on abdomen (Figure 14L). The longest seta is 3.25 mm long; the longest hastiseta is 0.5 mm long.

#### 3.4.17. Specimen 36 (PED 3663, Figure 15)

**Figure 15:**
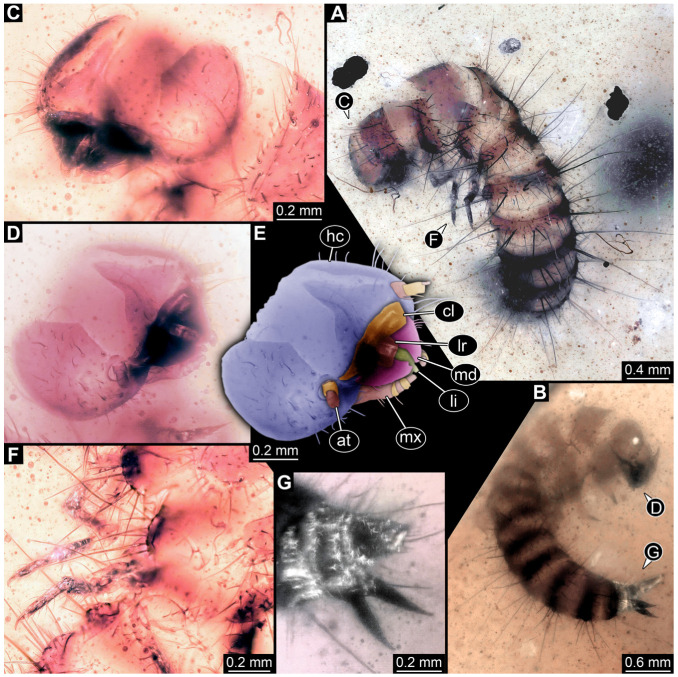
Specimen 36 (PED 3663), fossil larva of Dermestidae in Cretaceous Kachin Myanmar amber. (**A**) Habitus in dorsal view, thorax partially torn apart. (**B**) Habitus in lateral view. (**C**) Close-up of head in antero-lateral view. (**D**) Close-up of head in antero-dorsal view. (**E**) Color-marked version of (**D**). (**F**) Close-up of legs. (**G**) Posterior end of trunk with two urogomphi and pygopod. Abbreviations: at = antenna; cl = clypeus; hc = head capsule; li = labium; lr = labrum; md = mandible; mx = maxilla.

Single specimen preserved in Kachin Myanmar amber. Total body length around 4.6 mm. Specimen fragmentarily preserved, representing an exuvia (Figure 15A,B). Head hypognathous, with mouthparts directed ventrally (Figure 15C–E). Head capsule torn at the molting suture (Figure 15C–E). Both antennae discernible (Figure 15E), with three elements (antennomeres) each (Figure 15E). Antenna in total shorter than the head capsule. Head appendages partially accessible: heavily sclerotized mandibles; partial maxilla with palps (Figure 15E), with at least four elements (palpomeres) each; labium with small palps (Figure 15E). Thorax segments each with a pair of locomotory appendages (legs; Figure 15A,F). Each segment of trunk bears a dorsal transversal row of erect setae, diverse in length. Spicisetae not discernible. Hastisetae not discernible. Two downward-oriented urogomphi present at the trunk end (Figure 15B,G). Pygopod with apparent sclerotized pigmented ring discernible (Figure 15B,G). The longest seta is at least 1.3 mm long.

#### 3.4.18. Specimen 37 (PED 2926, Figure 16A–D)

**Figure 16:**
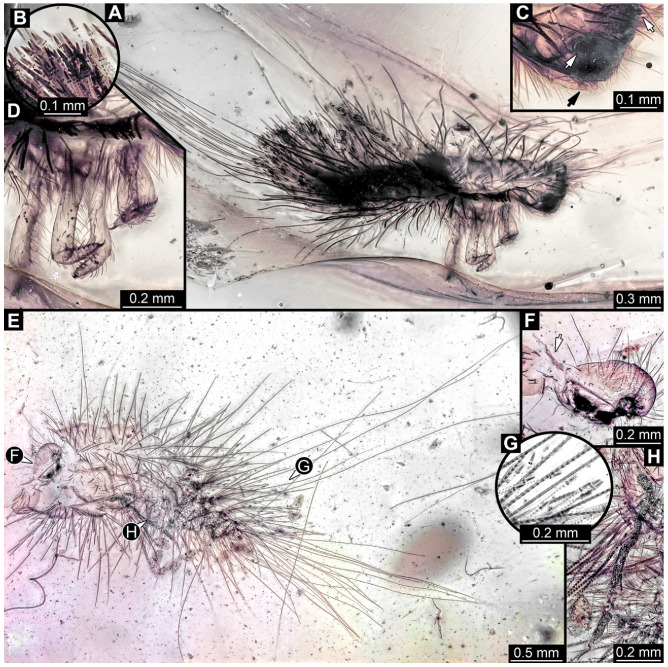
Fossil larvae of Dermestidae in Cretaceous Kachin Myanmar amber: (**A**–**D**) specimen 37 (PED 2926). (**A**) Habitus in lateral view. (**B**) Close-up of hastisetae. (**C**) Close-up of head with antennae (white arrows) and maxillary palps (black arrow); (**D**) Close-up of legs. (**E**–**H**) Specimen 38 (PED 3857). (**E**) Habitus in ventral view, anterior part of body torn apart. (**F**) Close-up of head with antenna (arrow) in posterior view. (**G**) Close-up of hastisetae. (**H**) Close-up of leg and spicisetae.

Single specimen preserved in Kachin Myanmar amber. Total body length around 1.6 mm. Specimen fragmentarily preserved, damaged, partly disarticulated, likely representing an exuvia. Head probably with mouthparts directed ventrally. Both antennae partially discernible (Figure 16C: white arrows), but exact number of elements are not discernible. Mouthparts partially discernible: maxillary palps (Figure 16C: black arrow), with at least four elements (palpomeres) each. Thorax segments each with a pair of locomotory appendages (legs; Figure 16D). Head and trunk bear sharp setae and spicisetae; spicisetae are of different lengths, some very long, but the total length of longest spicisetae is not accessible due to a crack in the amber (Figure 16A). Hastisetae present on the entire trunk, and especially dense on the posterior segments (Figure 16A,B). The longest seta is at least 1.6 mm long; the longest hastiseta is 0.65 mm long.

#### 3.4.19. Specimen 38 (PED 3857, Figure 16E–H)

Single specimen preserved in Kachin Myanmar amber. Total body length around 2 mm. Specimen only fragmentarily preserved, likely representing an exuvia. Head probably with mouthparts directed ventrally. One antenna discernible, with three elements (antennomeres; Figure 16F). Antenna in total shorter than head capsule. Specimen appears damaged at the thorax (Figure 16E). Thorax segments each with a pair of locomotory appendages (legs; Figure 16E). Head and trunk bear sharp setae and spicisetae (Figure 16H); posterior segments of abdomen bear some extremely long setae (Figure 16E), of up to 3.4 mm. Hastisetae discernible on trunk (Figure 16G). The longest hastiseta is 0.7 mm long.

#### 3.4.20. Specimen 39 (PED 0707, Figure 17A–D)

**Figure 17:**
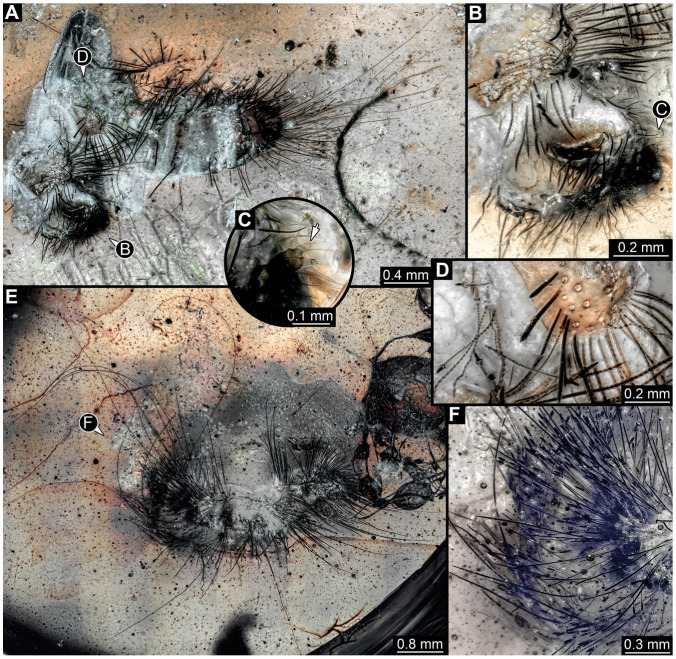
Fossil larvae of Dermestidae in Cretaceous Kachin Myanmar amber: (**A**–**C**) specimen 39 (PED 0707). (**D**,**E**) Specimen 40 (PED 0809). (**A**) Habitus, specimen damaged and partially covered in Verlumung. (**B**) Close-up on the head and prothorax. (**C**) Close-up on head with antenna (arrow). (**D**) Close-up on the thick setae and hastisetae. (**E**) Habitus in dorsal view, partially covered in Verlumung. (**F**) Close-up on the hastisetae.

Single specimen preserved in Kachin Myanmar amber. Length of discernible part of body around 3 mm. Specimen fragmentarily preserved, damaged, partly disarticulated, and covered in Verlumung (Figure 17A). Head probably with mouthparts directed ventrally, but no details discernible (Figure 17B). One antenna discernible, with at least three elements (antennomeres), and large sensory process (Figure 17C: arrow). Ultimate element (antennomere 3) thinner than rest. Antenna in total shorter than head capsule. No locomotory appendages (legs) discernible on thorax segments but presumed to be present (Figure 17A). Trunk and head bear sharp setae and spicisetae. A tuft of apparently smooth, extremely long setae on the posterior end of abdomen of up to 1.7 mm. Hastisetae discernible at posterior segments of the trunk (Figure 17A,B,D). The longest hastiseta is up to 0.5 mm long.

#### 3.4.21. Specimen 40 (PED 0809, Figure 17E,F)

Single specimen preserved in Kachin Myanmar amber. Length of discernible part of body around 3.7 mm long. Specimen appears to be completely preserved, but it is covered in Verlumung (Figure 17E). Head not accessible. No locomotory appendages (legs) discernible on thorax but presumed to be present (Figure 17E). Body bears sharp, long setae and spicisetae (Figure 17E). A tuft of sparse, smooth, extremely long setae is present on the posterior end of the abdomen, extending up to 4.6 mm. Hastisetae discernible (Figure 17F). The longest hastiseta is 0.5 mm long.

#### 3.4.22. Specimen 41 (PED 0647, Figure 18)

**Figure 18:**
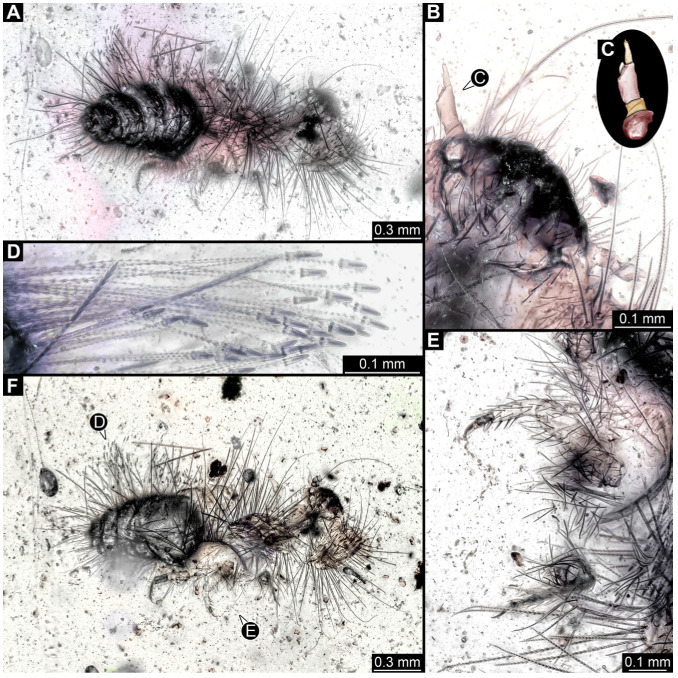
Specimen 41 (PED 0647), fossil larva in Cretaceous Kachin Myanmar amber: (**A**) Habitus in postero-lateral view, body partially torn apart. (**B**) Close-up of head. (**C**) Color-marked antenna. (**D**) Close-up of hastisetae. (**E**) Close-up of legs. (**F**) Habitus in lateral view.

Single specimen preserved in Kachin Myanmar amber. Total body length around 2 mm. Specimen fragmentarily preserved, appears damaged and disarticulated (Figure 18A,F). Head probably with mouthparts directed ventrally. Both antennae discernible (Figure 18B,C), with three discernible elements (antennomeres) each (Figure 18C); penultimate element (antennomere 2) with cone-shaped sensory process distolaterally. Antenna in total potentially as long as head capsule, but difficult to discern because head disarticulated and damaged. Mouthparts only partially discernible. Thorax very disarticulated and difficult to discern. Thorax segments each with a pair of locomotory appendages (legs; Figure 18A,E,F). Three elements of each appendage discernible, though presumably five elements are present. Head and trunk bear long setae up to 0.9 mm (Figure 18A), and spicisetae. Hastisetae discernible (Figure 18D). The longest seta is 0.9 mm long; the longest hastiseta is about 0.4 mm long.

#### 3.4.23. Specimen 42 (PED 1849, Figure 19)

**Figure 19:**
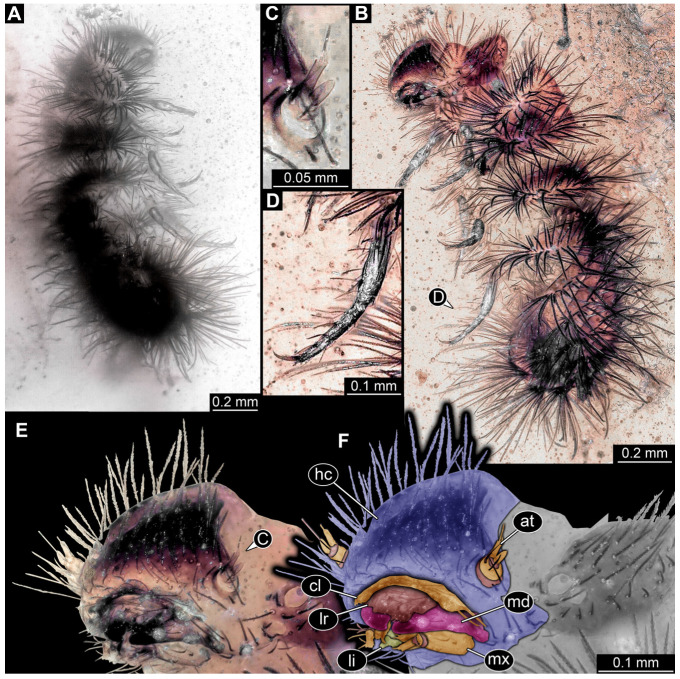
Specimen 42 (PED 1849), fossil larva in Cretaceous Kachin Myanmar amber. (**A**,**B**) Habitus in lateral view. (**C**) Close-up of antenna. (**D**) Close-up of leg. (**E**) Close-up of head. (**F**) Color-marked version of (**E**). Abbreviations: at = antenna; cl = clypeus; hc = head capsule; li = labium; lr = labrum; md = mandible; mx = maxilla.

Single specimen preserved in Kachin Myanmar amber. Total body length around 1.6 mm. Specimen appears slightly damaged and partly disarticulated (Figure 19A,B). Head probably with mouthparts directed ventrally. Both antennae discernible, with three elements (antennomeres) each (Figure 19C,F), and membrane between elements discernible; ultimate element (antennomere 3) thinner than rest; penultimate element (antennomere 2) with cone-shaped sensory process distolaterally, and slightly shorter than ultimate element (antennomere 3). Antenna in total shorter than head capsule. Clypeus and labrum parted by suture. Mouthparts partially discernible: heavily sclerotized mandibles; partial maxilla with palps with several elements (palpomeres); labium with small palps discernible (Figure 19E,F). Thorax segments presumably each with a pair of locomotory appendages (legs; Figure 19A,B). Four elements of appendages discernible (Figure 19D). Head and trunk bear long setae up to 0.4 mm (Figure 19B). Hastisetae not discernible.

#### 3.4.24. Specimen 43 (PED 3892, Figure 20)

**Figure 20:**
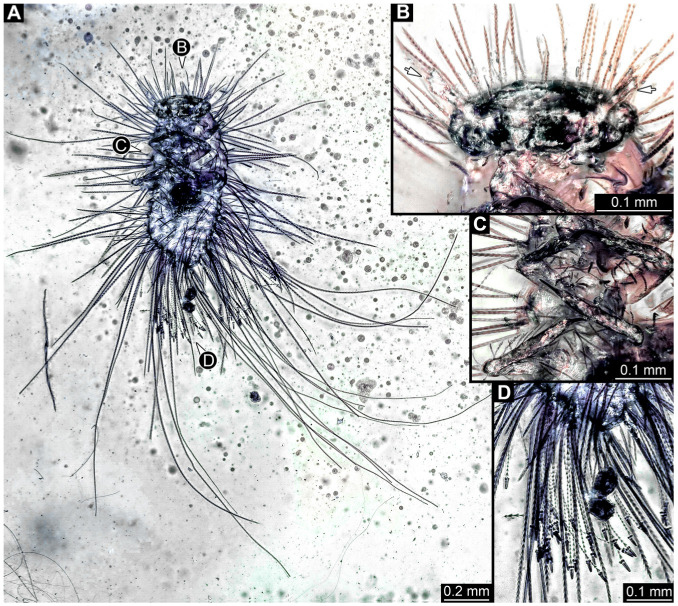
Specimen 43 (PED 3892), fossil larva in Cretaceous Kachin Myanmar amber. (**A**) Habitus in ventral view. (**B**) Close-up of head with antennae (arrows). (**C**) Close-up of legs. (**D**) Close-up of hastisetae and spicisetae.

Single specimen preserved in Kachin Myanmar amber. Total body length around 0.8 mm. Specimen appears to be completely preserved (Figure 20A). Head probably with mouthparts directed ventrally. Both antennae discernible (Figure 20B), with three elements (antennomeres) each (Figure 20B); penultimate element (antennomere 2) with cone-shaped sensory process distolaterally, about half as long as ultimate element (antennomere 3; Figure 20B: arrows). Antenna in total potentially as long as head capsule. Thorax segments each with a pair of locomotory appendages (legs; Figure 20A,C). Head and trunk bear long setae up to 1.6 mm (Figure 20A), and spicisetae. Hastisetae discernible (Figure 20D). The longest hastiseta is 0.4 mm long.

#### 3.4.25. Specimen 44 (PED 3926, Figure 21)

**Figure 21:**
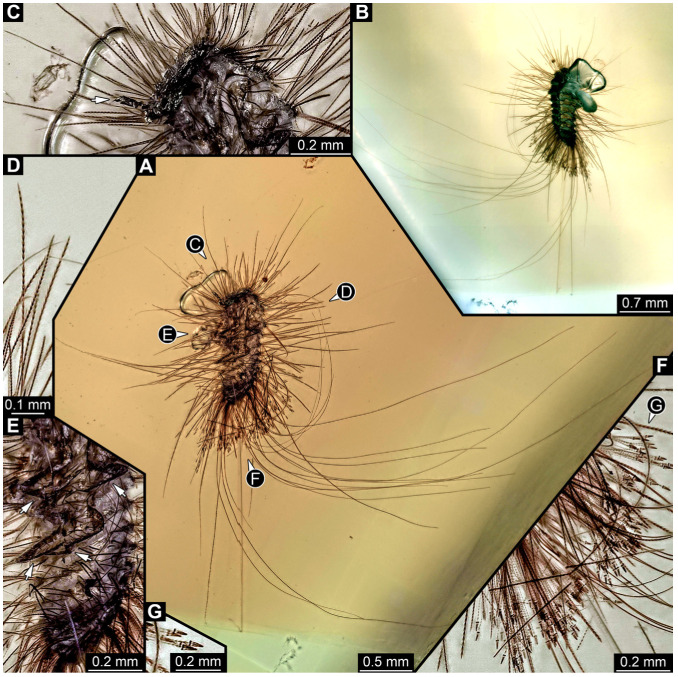
Specimen 44 (PED 3926), fossil larva in Cretaceous Kachin Myanmar amber. (**A**,**B**) Habitus lateral, in different light. (**C**) Close-up of head with antenna (arrow) in ventral view. (**D**) Close-up of spicisetae. (**E**) Close-up of legs (arrows) and trunk in ventral view. (**F**) Close-up of hastisetae of abdomen. (**G**) Close-up of the head of hastisetae.

Single specimen preserved in Kachin Myanmar amber. Total body length around 1.1 mm. Specimen appears to be completely preserved (Figure 21A,B). Head probably with mouthparts directed ventrally. Both antennae discernible, with three elements (antennomeres) each, but difficult to discern (Figure 21C: arrow). Ultimate element (antennomere 3) thinner than rest. Antenna in total potentially as long as head capsule. Mouthparts not discernible. Thorax segments each with a pair of locomotory appendages (legs; Figure 21A,E: arrows). Four elements of appendages discernible (Figure 21E). Head and trunk bear long setae up to 4.8 mm (Figure 21A) and spicisetae (Figure 21D). Hastisetae discernible (Figure 21F,G). The longest hastiseta is 0.7 mm long.

#### 3.4.26. Specimen 45 (PED 3917, Figure 22A,E,G)

**Figure 22:**
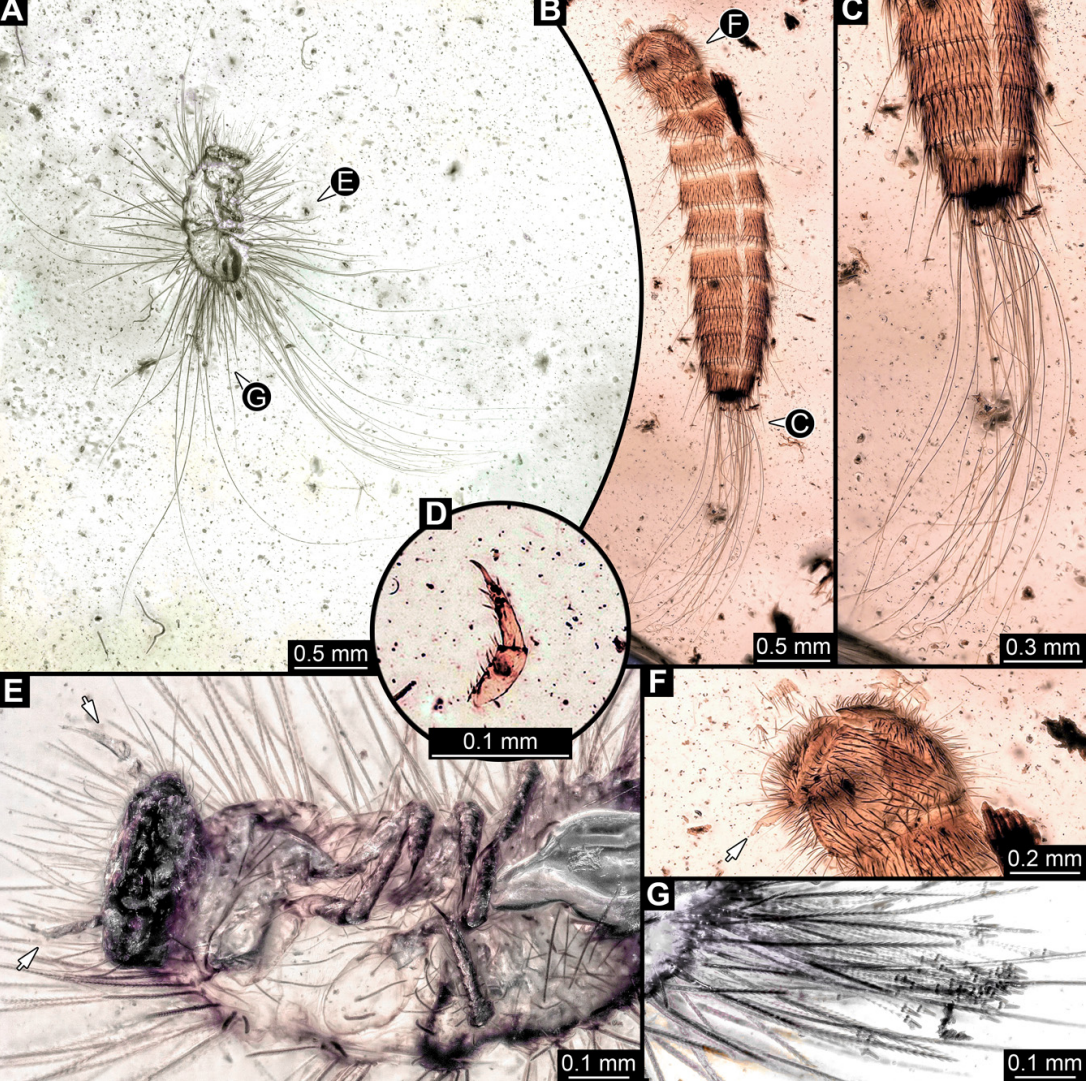
Fossil larvae of Dermestidae in Cretaceous Kachin Myanmar amber. (**A**,**E**,**G**) Specimen 45 (PED 3917). (**B**–**D**,**F**) Specimen 46 (PED 3705). (**A**) Habitus in ventro-lateral view. (**B**) Exuvia in dorso-lateral view, dorsally on head, pro- and mesothorax exuvia is torn apart at the suture. (**C**) Close-up of posterior part of abdomen and posterior brush of hairs. (**D**) Single incomplete leg. (**E**) Close-up of head with antennae (arrows) and thorax in ventral view. (**F**) Close-up of head with antenna (arrow). (**G**) Close-up of hastisetae on abdomen.

Single specimen preserved in Kachin Myanmar amber. Total body length around 1 mm. Specimen appears to be completely preserved but partially covered by Verlumung and bubbles (Figure 22A). Head probably with mouthparts directed ventrally. Both antennae discernible (Figure 22E: arrows), with three elements (antennomeres) each; ultimate element (antennomere 3) thinner than rest, tapering distally. Antenna in total potentially longer than head capsule. Mouthparts not discernible. Thorax segments each with a pair of locomotory appendages (legs; Figure 22A,E). Five elements of legs discernible (Figure 22E). Head and trunk bear long setae up to 2.8 mm (Figure 22A) and spicisetae. Hastisetae discernible (Figure 22A,G). The longest hastiseta is 0.5 mm long.

#### 3.4.27. Specimen 46 (PED 3705, Figure 22B–D,F)

Single complete specimen and an isolated appendage (potentially belonging to complete specimen) preserved in Kachin Myanmar amber. Total body length around 2.70 mm. Specimen appears to be completely preserved (Figure 22B), possibly representing an exuvia (head capsule and thorax segments torn dorsally on the suture). Trunk with dorsal longitudinal line discernible. Head probably with mouthparts directed ventrally. One antenna discernible (Figure 22F: arrow), with at least two elements (antennomeres, but difficult to discern proximally; penultimate element (antennomere 2) with potentially cone-shaped sensory process distolaterally, much shorter than subsequent element; ultimate element (antennomere 3) thinner than rest. Accessible part of antenna about half as long as head capsule. Mouthparts not discernible. No locomotory appendages (legs) discernible attached to body (Figure 22B). Single, isolated locomotory appendage (a leg) with three elements discernible (Figure 22D). Head and trunk bear long setae up to 2.2 mm (Figure 22B,C). Hastisetae not discernible.

#### 3.4.28. Specimen 47 (PED 3960, Figure 23)

**Figure 23:**
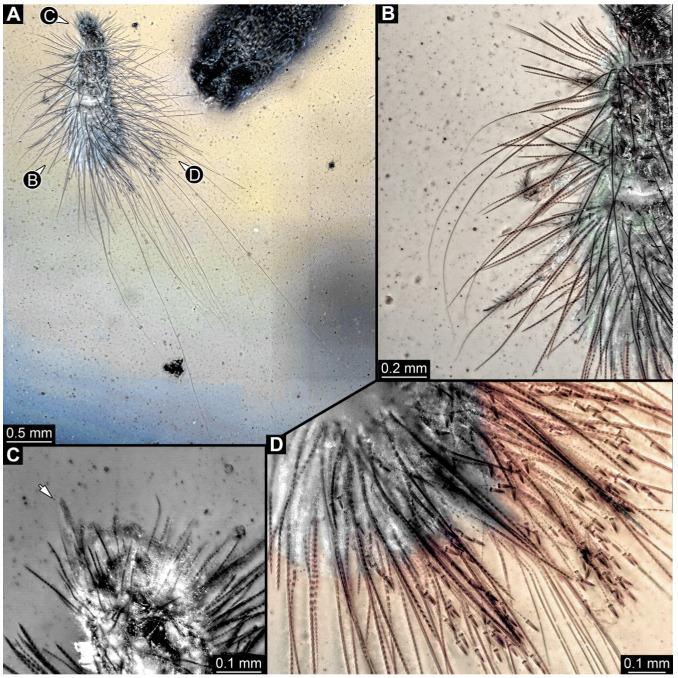
Specimen 47 (PED 3960), fossil larva in Cretaceous Kachin Myanmar amber. (**A**) Habitus in dorso-lateral view. (**B**) Close-up of legs and spicisetae. (**C**) Close-up of head with antenna (arrow). (**D**) Close-up of the hastisetae.

Single specimen preserved in Kachin Myanmar amber. Total body length around 1.75 mm. Specimen appears completely preserved (Figure 23A). Head probably with mouthparts directed ventrally. A single antenna discernible (Figure 23C: arrow), exact number of elements (antennomeres) not discernible (Figure 23C). Antenna in total shorter than head capsule. Mouthparts not discernible. Thorax segments each with a pair of locomotory appendages (legs; Figure 23B). Head and trunk bear long setae and spicisetae (Figure 23B). Hastisetae discernible (Figure 23D). The longest seta is about 3.9 mm long, the longest hastiseta is about 0.5 mm long.

#### 3.4.29. Specimen 48 (PED 3961, Figure 24)

**Figure 24:**
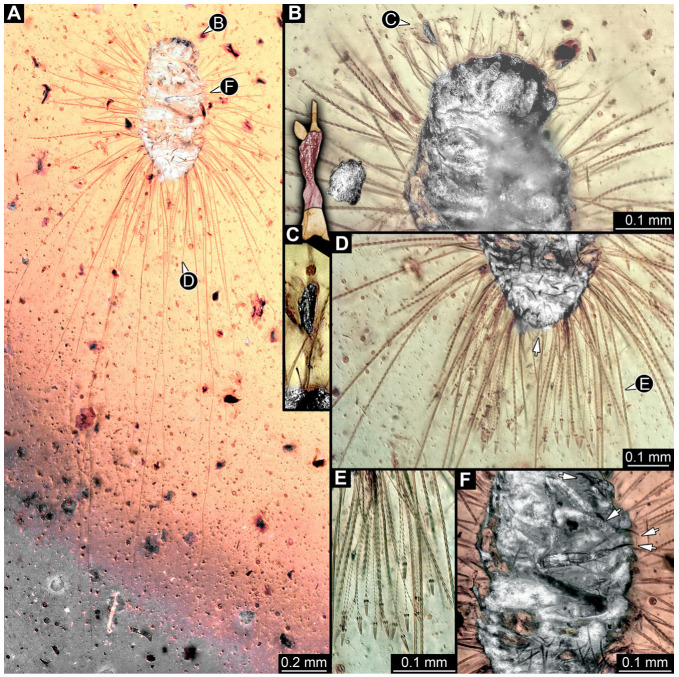
Specimen 48 (PED 3961), fossil larva in Cretaceous Kachin Myanmar amber. (**A**) Habitus in ventral view. (**B**) Close-up of head in ventral view. (**C**) Close-up of antenna and its color-marked version (200% enlarged from (**B**)). (**D**) Close-up of posterior trunk with short setae at the trunk end (arrow). (**E**) Close-up of hastisetae. (**F**) Close-up of legs with tarsungula (arrows).

Single specimen preserved in Kachin Myanmar amber. Total body length around 0.6 mm. Specimen appears completely preserved but partially covered by Verlumung and other syn-inclusions (Figure 24A). Head probably with mouthparts directed ventrally. One antenna discernible (Figure 24B,C), with three elements (antennomeres, Figure 24C), but difficult to discern; ultimate element (antennomere 3) thinner than rest; penultimate element (antennomere 2) with cone-shaped sensory process distolaterally, about half as long as ultimate element. Antenna in total potentially longer than head capsule. Mouthparts not discernible. Trunk differentiated into thorax and abdomen. Thorax segments each with a pair of locomotory appendages (legs; Figure 24F: arrows). Head and trunk bear long setae (Figure 24A) and spicisetae (Figure 24B,D). Hastisetae discernible (Figure 24E). The longest seta is about 2.25 mm long, the longest hastiseta is about 0.35 mm long.

#### 3.4.30. Specimen 49 (BUB3346, Figure 9E–H)

Single specimen preserved in Kachin Myanmar amber. Total body length around 1.2 mm. Specimen only fragmentarily preserved, likely representing an exuvia (Figure 9E). Head probably with mouthparts directed ventrally. One antenna discernible (Figure 9F: arrow), exact number of elements (antennomeres) not discernible. Antenna shorter than head capsule. Mouthparts partially discernible; heavily sclerotized mandibles discernible (Figure 9F). Thorax segments each with a pair of locomotory appendages (legs; Figure 9E: white arrow). Head bears moderately short fine setae; trunk bears long fine setae up to 1.2 mm long, sharp setae, and spicisetae; trunk end bears a brush of long setae posteriorly (Figure 9E: black arrow). Hastisetae discernible, both on thorax (Figure 9G) and on posterior part of abdomen (Figure 9H). The longest hastiseta is about 0.25 mm long.

#### 3.4.31. Specimen 50 (BUB 3184, Figure 25A,B)

**Figure 25:**
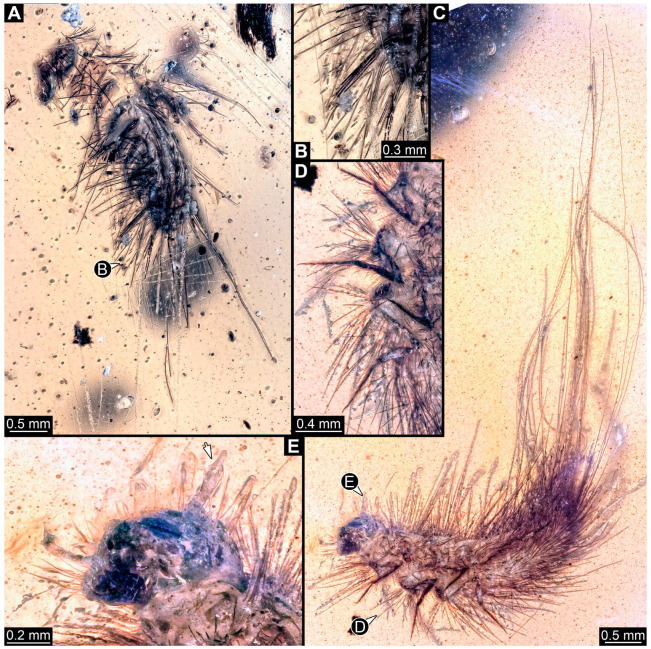
Fossil larvae in Cretaceous Kachin Myanmar amber. (**A**,**B**) Specimen 50 (BUB 3184). (**A**) Habitus in dorsal view, specimen incomplete, head and thorax torn apart. (**B**) Close-up of hastisetae. (**C**–**E**) Specimen 51 (BUB 3353). (**C**) Habitus in lateral view. (**D**) Close-up of legs. (**E**) Close-up of head with antennae (arrow).

Single specimen preserved in Kachin Myanmar amber. Total body length around 2.9 mm. Specimen only fragmentarily preserved (Figure 25A), likely representing an exuvia (head capsule and thorax segments torn dorsally on the suture). Details of the head are not observable. Locomotory appendages (legs) of thorax segments not discernible but presumed to be present. Head and trunk bear long setae and spicisetae. Hastisetae discernible (Figure 25B). The longest seta is about 2.2 mm long; the longest hastiseta is about 1 mm long.

#### 3.4.32. Specimen 51 (BUB3353, Figure 25C–E)

Single specimen preserved in Kachin Myanmar amber. Total body length around 3.6 mm. Specimen is completely preserved but partially covered by Verlumung and bubbles (Figure 25C). Head probably with mouthparts directed ventrally, but no details discernible. Both antennae discernible (Figure 25C,E: arrow); antenna with three elements (antennomeres); distal element (antennomere 3) thinner than rest (Figure 25C,E). Antenna in total possibly as long as head capsule. Thorax segments each with a pair of locomotory appendages (legs; Figure 25D). Head and trunk bear long setae and spicisetae. Hastisetae discernible. The longest seta is at least 6.75 mm long; the longest hastiseta is about 1.6 mm long.

#### 3.4.33. Specimen 52 (PED 4043, Figure 26)

**Figure 26:**
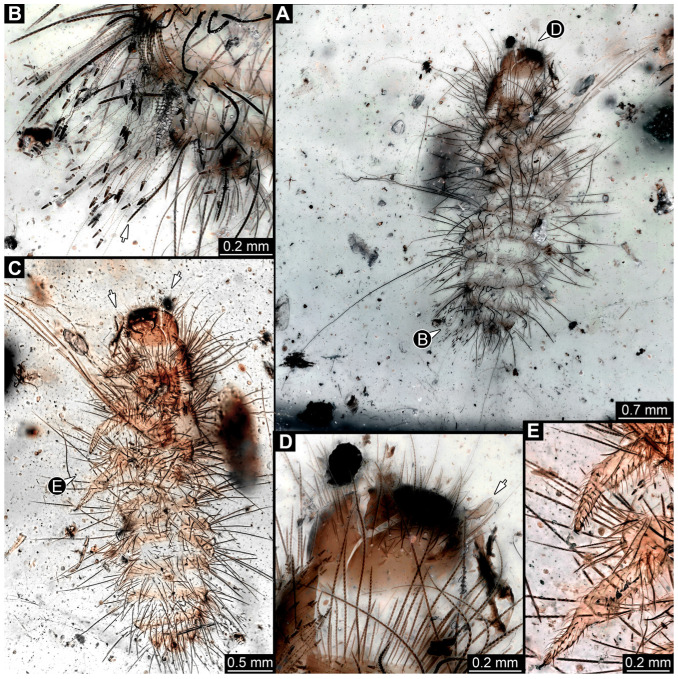
Specimen 52 (PED 4043), fossil larva in Cretaceous Kachin Myanmar amber. (**A**) Exuvia in dorsal view, dorsally on head, thorax and several abdomen tergites exuvia is torn apart at the suture. (**B**) Close-up of hastisetae and spiciseta (arrow). (**C**) Exuvia in ventral view, head with antennae (arrows). (**D**) Close-up of head with antenna (arrow). (**E**) Close-up of legs.

Single specimen preserved in Kachin Myanmar amber. Total body length around 3.75 mm. Specimen appears damaged and disarticulated, likely representing an exuvia (Figure 26A,C). Head probably with mouthparts directed ventrally. One antenna discernible (Figure 26C,D: arrows), with three elements (antennomeres); penultimate element (antennomere 2) with broad but short sensory process (Figure 26D); ultimate element (antennomere 3) thinner than rest (Figure 26C). Antenna in total shorter than head capsule. Mouthparts not discernible. Thorax segments each with a pair of locomotory appendages (legs; Figure 26E). Head and trunk bear long setae and spicisetae (Figure 26B: arrow). Hastisetae discernible (Figure 26B). The longest seta is at least 3 mm long, the longest hastiseta is about 0.65 mm long.

#### 3.4.34. Specimen 53 (PED 4051, Figure 27)

**Figure 27:**
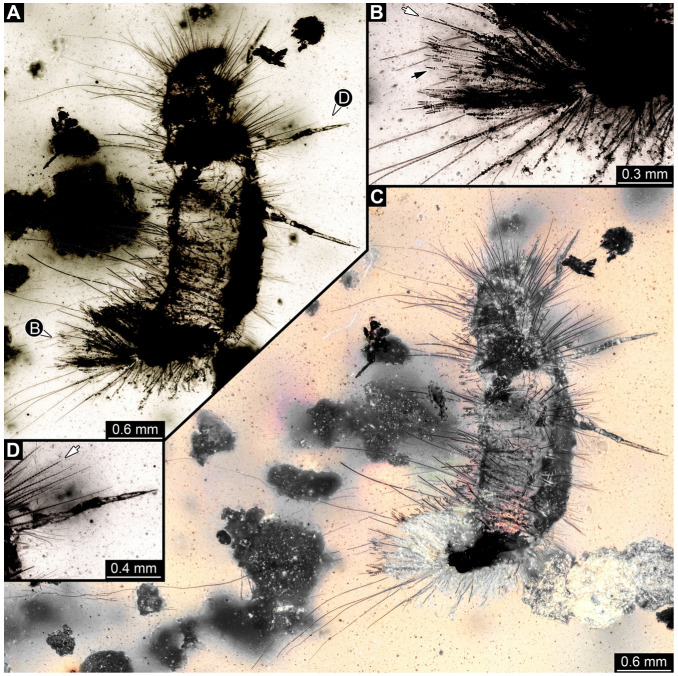
Specimen 53 (PED 4051), fossil larva in Cretaceous Kachin Myanmar amber. (**A**) Grayscale image of habitus in lateral view. (**B**) Close-up of posterior brush of hastisetae (black arrow) surrounded by spicisetae (white arrow). (**C**) Habitus in lateral view. (**D**) Close-up of leg, spiciseta discernible (arrow).

Single specimen preserved in Kachin Myanmar amber. Total body length around 3.6 mm. Specimen is completely preserved but partially covered by Verlumung and syn-inclusions (Figure 27A,C). Head probably with mouthparts directed ventrally, but no details discernible. Antennae not discernible. Thorax segments each with a pair of locomotory appendages (legs; Figure 27A,C). Head and trunk bear long setae and spicisetae (Figure 27B,D: white arrows). Hastisetae discernible (Figure 27B: black arrow). The longest seta is at least 5 mm long; the longest hastiseta is about 2.05 mm long.

#### 3.4.35. Specimen 54 (PED 4148, Figure 28A–D)

**Figure 28:**
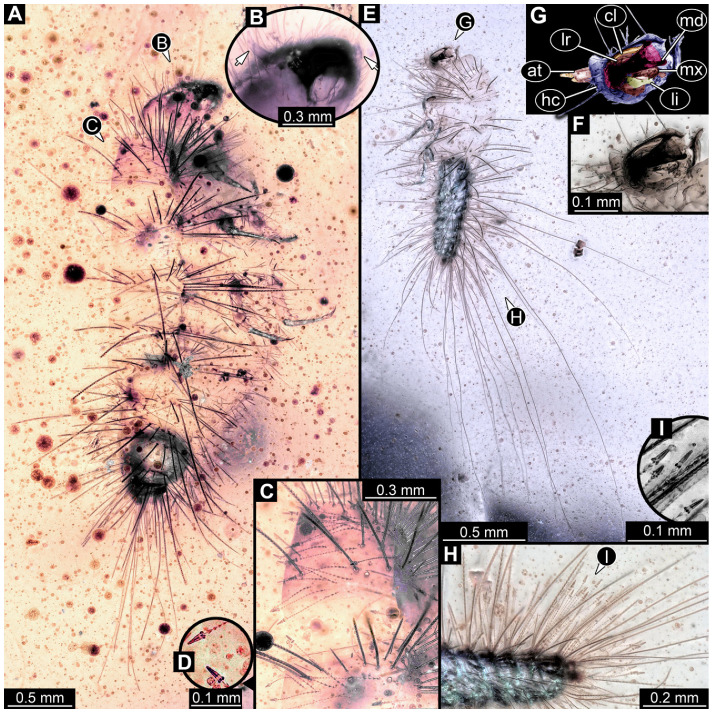
Fossil larvae in Cretaceous Kachin Myanmar amber. (**A**–**D**) Specimen 54 (PED 4148). (**E**–**I**) Specimen 55 (PED 4168). (**A**) Habitus in lateral view. (**B**) Close-up of head with antennae (arrows). (**C**) Close-up of pro- and mesothorax with hasti- and spicisetae. (**D**) Close-up of heads of hastisetae. (**E**) Habitus in ventro-lateral view. (**F**) Close-up of head. (**G**) Color-marked version of (**F**). (**H**) Close-up of posterior part of abdomen with hastisetae. (**I**) Close-up of heads of hastisetae, in black and white. Abbreviations: at = antenna; cl = clypeus; hc = head capsule; li = labium; lr = labrum; md = mandible; mx = maxilla.

Single specimen preserved in Kachin Myanmar amber. Total body length around 3.6 mm. Specimen is completely preserved but partially covered by other inclusions (Figure 28A). Head probably with mouthparts directed ventrally. Both antennae discernible (Figure 28B: arrows), with three elements (antennomeres); ultimate element (antennomere 3) thinner than rest. Antenna in total shorter than head capsule. Thorax segments each with a pair of locomotory appendages (legs), but only fragmentarily preserved (Figure 28A). Head and trunk bear long setae and spicisetae. Hastisetae discernible (Figure 28C,D). The longest seta is at least 5.5 mm long; the longest hastiseta is about 1 mm long.

#### 3.4.36. Specimen 55 (PED 4168, Figure 28E–I)

Single specimen preserved in Kachin Myanmar amber. Total body length around 1.35 mm. Specimen appears damaged and partly disarticulated (Figure 28E). Head probably with mouthparts directed ventrally. One antenna discernible, with at least two elements (antennomeres); ultimate element thinner than rest. Clypeus and labrum discernible, parted by suture. Mouthparts partially discernible (Figure 28G): heavily sclerotized mandibles; partial maxilla, maxillary palp with at least three elements (palpomeres); partial labium (Figure 28F,G). Thorax segments each with a pair of locomotory appendages (legs; Figure 28E). Head and trunk bear long setae and spicisetae. Hastisetae discernible (Figure 28H,I). The longest seta is about at least 1.9 mm long; the longest hastiseta is 0.3 mm long.

#### 3.4.37. Specimen 56 (PED 4406, Figure 29A,C–E)

**Figure 29:**
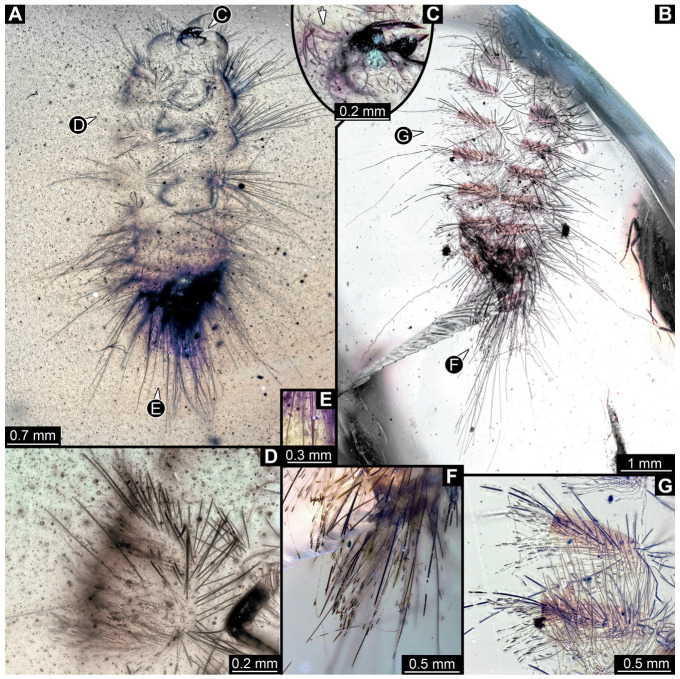
Fossil larvae in Cretaceous Kachin Myanmar amber. (**A**,**C**–**E**) Specimen 56 (PED 4406). (**B**,**F**,**G**) Specimen 57 (PED 4409). (**A**) Habitus in ventral view. (**B**) Exuvia in dorsal view. (**C**) Close-up of head with antenna (arrow). (**D**) Close-up of thorax with hasti- and spicisetae. (**E**) Close-up of hastisetae from abdomen. (**F**) Close-up of hasti- and spicisetae from posterior abdomen. (**G**) Close-up of hasti- and spicisetae from anterior abdomen.

Single specimen preserved in Kachin Myanmar amber. Total body length around 4.25 mm. Specimen only fragmentarily preserved, likely representing an exuvia (Figure 29A). Head probably with mouthparts directed ventrally. One antenna discernible (Figure 29A,C: arrow), with three elements (antennomeres); ultimate element (antennomere 3) thinner than rest. Antenna in total shorter than head capsule. Mouthparts partially discernible: heavily sclerotized mandibles, partial maxilla (Figure 29C). Thorax segments each with a pair of locomotory appendages (legs; Figure 29A). Head bears moderately short setae, trunk with areas of longer sharp setae and spicisetae (Figure 29A,D). Hastisetae discernible both on thorax (Figure 29D) and on posterior part of abdomen (Figure 29E). The longest seta is at least 1.65 mm long; the longest hastiseta is about 0.6 mm long.

#### 3.4.38. Specimen 57 (PED 4409, Figure 29B,F,G)

Single specimen preserved in Kachin Myanmar amber. Specimen fragmentarily preserved, likely representing an exuvia (Figure 29B). Head not preserved. Only partial trunk of specimen preserved, and length of preserved part 5.4 mm (Figure 29B). Only posterior part of thorax preserved, with a single locomotory appendage discernible (a leg; Figure 29B). Trunk bears areas of long setae and spicisetae (Figure 29F). Hastisetae discernible on each preserved segment of trunk (Figure 29B,F,G). The longest seta is at least 3.1 mm long; the longest hastiseta is at least 1.2 mm long.

#### 3.4.39. Specimen 58 (PED 4379, Figure 30A–C)

**Figure 30:**
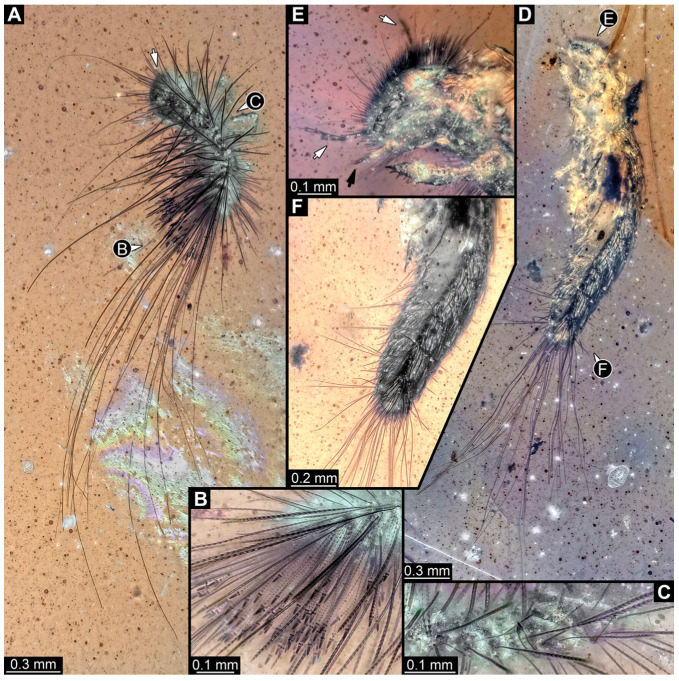
Fossil larvae in Cretaceous Kachin Myanmar amber. (**A**–**C**) Specimen 58 (PED 4379). (**A**) Habitus in lateral view, head with antenna (arrow). (**B**) Close-up of hasti- and spicisetae of abdomen. (**C**) Close-up of two hasti- and many spicisetae of thorax. (**D**–**F**) Specimen 59 (PED 4380). (**D**) Habitus in ventro-lateral view. (**E**) Close-up of head with antennae (white arrows) and mouthparts (black arrow). (**F**) Close-up of abdomen with posterior tuft of setae and a leg.

Single specimen preserved in Kachin Myanmar amber. Total body length around 0.9 mm. Specimen is completely preserved (Figure 30A). Head with mouthparts directed ventrally (Figure 30A). One antenna discernible (Figure 30A: arrow), exact number of elements (antennomeres) not discernible. Antenna in total shorter than head capsule. Thorax segments each with a pair of locomotory appendages (legs; Figure 30A). Head and trunk bear long setae and spicisetae (Figure 30C). Hastisetae discernible (Figure 30B). The longest seta is at least 2.5 mm long; the longest hastiseta is about 0.3 mm long.

#### 3.4.40. Specimen 59 (PED 4380, Figure 30D–F)

Single specimen preserved in Kachin Myanmar amber. Total body length around 2.1 mm. Specimen is completely preserved but partially covered by Verlumung and syn-inclusions (Figure 30D). Head with mouthparts directed ventrally (Figure 30E). Both antennae discernible (Figure 30D,E: white arrows), with three elements (antennomeres); distal element (antennomere 3) thinner than rest. Antenna almost as long as head capsule. Mouthparts partially discernible: heavily sclerotized mandibles, partial maxilla, maxillary palp with at least two elements (palpomeres; Figure 30E: black arrow). Thorax segments each with a pair of locomotory appendages (legs; Figure 30D). Head bears moderately short, thick setae; trunk bears longer, thick setae laid close to body (Figure 30F), and brush of long setae posterior to trunk end (Figure 30D). Hastisetae not discernible. The longest seta is at least 1.5 mm long.

### 3.5. Morphometric Analyses of Dermestidae

Although the results of the analyses on the two datasets (i.e., the complete dataset and the dataset without fragmented specimens) are seemingly similar, the results of the Kruskal tests and Dunn post hoc tests show differences. The dataset including fragmented specimens differs by showing the following: (1) Eocene specimens have significantly shorter setae than Cretaceous specimens, and (2) there are no significant differences in body length nor absolute length of hastisetae between the time slices (Supplementary Table S3).

**Figure 31:**
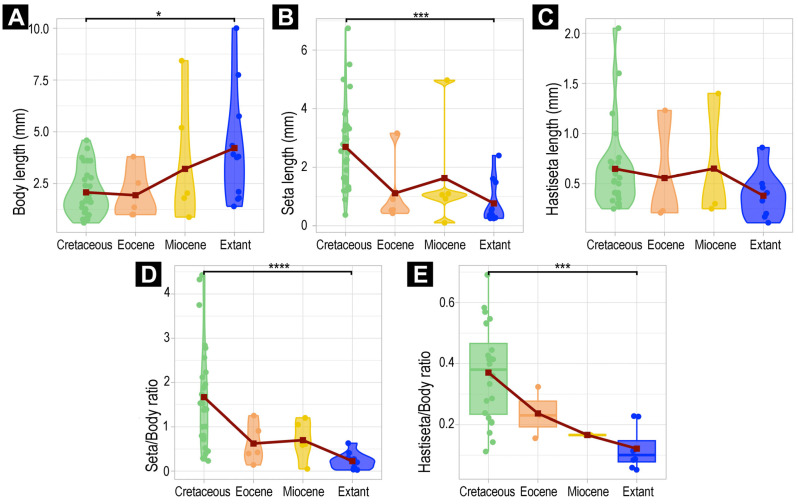
Violin plots and a boxplot explaining the variation in the measurements of extant and fully preserved fossil larvae of Dermestidae, and their hastisetae. (**A**–**D**) Violin plots of larvae of Dermestidae, from different ages. (**A**) Violin plot of body length. (**B**) Violin plot of seta length. (**C**) Violin plot of hastisetae length. (**D**) Violin plot of seta–body ratio. (**E**) Boxplot hastiseta–body length ratio of larvae of Dermestidae, from different ages. Asterisks represent *p*-values: * = *p* < 0.05, *** = *p* < 0.001, **** = *p* < 0.0001.

The dataset without fragmented specimens shows that there are significant differences between extant and Cretaceous specimens (Dunn tests *p* < 0.05; Figure 31, Supplementary Table S3), except for the absolute length of the hastisetae (Kruskal–Wallis *p* = 0.1857). In comparison to extant larvae of Dermestidae, Cretaceous larvae have (1) smaller body (*p* < 0.05), (2) significantly longer setae (*p* < 0.001), (3) significantly higher setae–body ratios (*p* < 0.0001), and finally (4), significantly higher hastisetae–body ratios (*p* < 0.001).

### 3.6. Morphometric Analyses of Megatominae

**Figure 32:**
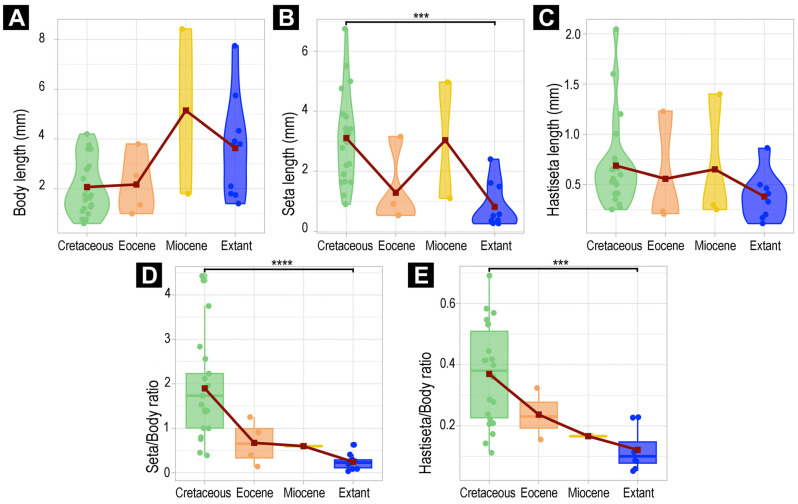
Violin plots and boxplots explaining the variation in the measurements of extant and fully preserved fossil larvae of Megatominae, and their hastisetae. (**A**–**C**) Violin plots of larvae of Megatominae, from different ages. (**A**) Violin plot of body length. (**B**) Violin plot of seta length. (**C**) Violin plot of hastisetae length. (**D**,**E**) Boxplots of larvae of Megatominae, from different ages. (**D**) Boxplot of seta/body ratio. (**E**) Boxplot of hastiseta/body length ratio. Asterisks represent *p*-values: *** = *p* < 0.001, **** = *p* < 0.0001.

In the Megatominae dataset (Figure 32), only the absolute lengths of the hastisetae showed no difference through time (Kruskal–Wallis *p* = 0.1337; Figure 32C). Despite being significant, the Dunn post hoc test also revealed no significant difference in body lengths over time (Supplementary Table S3). Regarding the other measurements, absolute and relative lengths significantly differed over time (Kruskal tests *p* < 0.05; Figure 32A,B,D,E).

Similarly to the dataset without fragmented larvae, Cretaceous larvae are again significantly different from extant larvae (Figure 32). Cretaceous larvae have (1) longer setae (*p* < 0.001), (2) higher setae–body ratios (*p* < 0.0001), and (3) higher hastisetae–body ratios (*p* < 0.01) compared to extant larvae.

## 4. Discussion

### 4.1. General Identity of Specimens: Larvae of Dermestidae

Most of the new specimens display a spindle-shaped body, which is sometimes more cylindrical than flattened, often coupled with a subglobular, hypognathous head, with mouthparts oriented ventrally. The head is either slightly retracted or protracted, and a distinct molting suture (referred to as the “epicranial suture”) is visible in several specimens (Figure 14C, Figure 15A–C and Figure 26D), appearing either intact or ruptured, indicative of larval molting events. Additionally, several specimens exhibit a prominent labrum, obviously separated from clypeus by a suture and an antenna with three elements (antennomeres). In specimens with accessible mouthparts, strongly sclerotized mandibles are discernible. In addition, in some specimens, maxillae with maxillary palps (with three or four palpomeres) and two endites (lacinia and galea) are discernible, as well. Each specimen bears numerous setae of varying lengths, both simple and specialized forms, known as spicisetae and hastisetae. These setal morphologies, along with the aforementioned traits, are consistent with modern larvae of Dermestidae, commonly referred to as skin, larder, or carpet beetles (Figure 1). However, a single specimen (OU 33636.3) exhibits a morphology of maxillary endites that is not known from the literature on extant specimens of Dermestidae. Therefore, we suggest that this specimen is either a larva of Dermestidae with an extinct morphology or a larva of a closely related group of Bostrichoidea.

### 4.2. Identity of Specimens Within Dermestidae: Possible Larvae of Orphilinae, Dermestinae, Trinodinae, Attageninae and Megatominae

Although some fossil specimens lack sufficient observable features for precise identification, many can be confidently identified to at least Dermestidae. Several specimens remain unidentified, likely due to damage sustained prior to entrapment in the resin or during the fossilization process itself, such as degradation, bubble formation, or the presence of syn-inclusions. An identification within Dermestidae can often be made based on the presence or absence of specialized setae with arrowhead tips, known as hastisetae. Within our fossil sample, several specimens lack hastisetae. A single incomplete larva, specimen 9 (OU 33636.3; Figure 7 and Figure 8), lacks hastisetae, but we cannot be completely sure these were not present on the posterior trunk prior to the sustained damage. Furthermore, an additional seven complete larvae do not bear hastisetae: specimen 28 (TMP 96.9.366; Figure 10B), specimen 29 (TMP 96.9.393a; Figure 10D), specimen 30 (TMP 96.9.393b, Figure 10F), specimen 36 (PED 3663; Figure 15), specimen 42 (PED 1849; Figure 19), specimen 46 (PED 3705; Figure 22B), and specimen 59 (PED 4380; Figure 30D). All other specimens display hastisetae on at least some body segments.

#### 4.2.1. Larvae of Morphotype 1

Specimen 9 (OU 33636.3; Figure 7 and Figure 8) has only a partially preserved body, yet its morphology differs strongly from the rest of the new specimens. The partial body seems cylindrical, much like the body of the extant larvae of *Orphilus* (Orphilinae, Dermestidae) [26], and it bears very short and spike-like setae that are not dense, similar to the setae of the larva of *Orphilus niger* Rossi, 1790 (their figure 1) [107]. However, the setae that the new specimen bears are not orientated posteriorly, remaining close to the body surface with their whole length, as seen in some larvae of Attageninae (Dermestidae) [108], but the setae are rather orientated perpendicular to the body surface. Additionally, the globular head (in lateral and anterior view), with ventrally positioned mouthparts, strongly resembles the heads of larvae of Dermestidae (i.e., their figure 3E–H) [26]. One antenna of specimen 9 is well visible, and it has three elements and a cone-like sensory process. The most distal element is slender and bears a strong seta at its distal tip. In comparison to the antenna of the larva of *O. niger* (their figure 7) [107], specimen 9 has a much longer distal element of the antenna (Figure 7B–E). However, the similarity in shape of other elements of antenna and the sensory process between the new specimen and the antenna of larva of *O. niger* is obvious. Additionally, the orientation of the antennae of this specimen differs from the orientation of antennae in all of the other new specimens. The antennae of specimen 9 are directed straight anteriorly (Figure 8C,D), whereas the antennae of other specimens are oriented antero-laterally. In Kiselyova & McHugh (their figure 7E–H) [26], larvae of *Orphilus* exhibit the same orientation of antennae as the new specimen 9. Extant larvae of *Orphilus* often have a single bump-like tubercle on the frontal part of their head capsule medially. Unfortunately, this feature is not accessible if present in our specimen due to the fissures of the cuticle and the syn-inclusions that surround it. Nevertheless, the clypeo-labral suture parting the clypeus from the labrum is discernible. Such a movable labrum is also a feature seen in many larvae of Dermestidae. The mouthparts are well-preserved, with mandibles, maxillae, and labium all partially observable. The distal tips of heavily sclerotized mandibles are visible, each with two teeth, a feature known from extant larvae of *Orphilus*. The maxillary palps of the new specimen have three elements (Figure 8A,B), just like the maxillary palps of extant larvae of Dermestidae. Interestingly, the maxillary endites (Figure 8B, Supplementary Figure S3) of the new specimen differ from the figured maxillary endites of both the larva of *O. niger* (their figure 3) [107] and the larva of *O. subnitidus* LeConte, 1861 (their figure 11E,F) [26]. The endites of the new specimen look together almost semi-circular in anterior view, with a very setose surface (Figure 8A,B). The numerous setae on the more proximal endite (lacinia) are large and flat (Figure 8A,B), but no bidentate hook-like structures are present, as in larvae of *O. subnitidus* (their figure 11E,F) [26]. Therefore, these morphological features resemble more the details of maxillary endites of larvae of *Endecatomus rugosus* Randall, 1838 (their figure 11C) [26] than the maxillary endites of larvae of *Orphilus*. *Endecatomus rugosus* is a species of Endecatomidae that, like Dermestidae, is part of a larger group Bostrichoidea. Therefore, we suggest that this fossil specimen is either a larva of Dermestidae, more specifically a larva of Orphilinae with an extinct morphology, or a representative of a closely related group, such as Endecatomidae.

However, we do suggest this identification with caution, since the line separating the two endites of maxilla (Figure 8A,B) is thin and there is a possibility that what we interpreted as lacinia and galea is actually a single mala with an artifact in the shape of a line (we provide additional raw scans and scan renderings of the area in Supplementary Figure S3). If this would be the case, we would no longer be able to identify this specimen as a representative of Bostrichoidea but rather as a possible representative of another large group, such as Cucujiformia.

#### 4.2.2. Larva of Morphotype 2

Specimen 36 (PED 3663; Figure 15) has a unique morphology among the new fossils figured in this publication. The specimen lacks both hastisetae and spicisetae but possesses other identifying features specific to certain ingroups of Dermestidae. Specimen 36 exhibits a molting suture, the antennae with no cone-shaped sensory process, and maxillary palps of the larvae of *Dermestes* [26]. Especially prominent are the two distinctive, downward-curved urogomphi on the trunk end of the specimen (Figure 15B,G). Curved urogomphi are diagnostic of the ingroups *Dermestes* and *Thorictodes* Reitter, 1875. Additionally, the fossil exhibits a partially sclerotized pygopod. Together, these traits–downward-curved urogomphi and a sclerotized pygopod ring—correspond to characteristics of extant larvae of *Dermestes*, such as *Dermestes lardarius* Linnaeus, 1758 (their figure 34(4,7,3,a)) [109]. Consequently, we propose that specimen 36 is a fossil representative of Dermestinae.

#### 4.2.3. Larvae of Morphotype 3

New specimen 42 (PED 1849; Figure 19) is another larva lacking hastisetae, but it bears rows of robust spicisetae on each tergite that radiate outward, forming circumferential rings of setae similar to the arrangement observed in larvae of Dermestidae, especially of *Apsectus hispidus* Melsheimer, 1844 (their figure 3J) [108] and in larvae of *Thylodrias* Motschulsky, 1839 [110]. Additional comparisons to larvae of *Thylodrias* depicted by Böving and Craighead (pl. 90H) [111] and Beal (their figure 34(4,7,8), p. 436) [109] reveal notable morphological similarities. While the antenna of specimen 42 (PED 1849; Figure 19C) does not precisely match the antennae of the larva of *Thylodrias* in Böving and Craighead [111], it closely resembles those of a larva of *Apsectus hispidus* (their figure 2M,N) [108]. Although some aspects of larvae of *Trinodes* [112] resemble the morphology of specimen 42, the absence of hastisetae, particularly the kind of hastisetae found in modern larvae of *Trinodes* [26], does not support the interpretation of this new specimen as a representative of *Trinodes.* However, the specimen is likely a larva of Trinodinae. Interestingly, another fossil larva of Trinodinae was already described by Kadej and Háva [65]; however, it was from Eocene Baltic amber. Three new specimens preserved in Canadian amber, specimen 28 (TMP 96.9.366), specimen 29 (TMP 96.9.393a), and specimen 30 (TMP 96.9.393b), have their setae organized in circumferential rings that radiate outwards (Figure 10B,D,F), similar to specimen 42 (PED 1849). Both specimen 29 (TMP 96.9.393a) and specimen 30 (TMP 96.9.393b) have short and stubby antennae with a thinner and longer ultimate element (Figure 10E,G), also similar to the antennae of *Apsectus hispidus* (their figure 2M,N) [108]. However, due to the fragmentation or partial concealment of the three new specimens, not all morphological features are discernible, and therefore, we can only identify these specimens as larvae of Dermestidae.

#### 4.2.4. Larvae of Morphotype 4

Larvae of this morphotype do not bear hastisetae on their body but do bear short simple setae that lay close to the body and a long terminal brush of simple setae. Among the new fossils, two specimens represent this morphotype: specimen 46 (PED 3705; Figure 22B) and specimen 59 (PED 4380; Figure 30D). According to Klausnitzer [110], larvae of Dermestidae that lack urogomphi and a pygopod, yet bear a terminal brush of simple setae, may be larvae of either *Attagenus* Latreille, 1802 (Attageninae) or *Thylodrias* (Trinodinae). Both specimen 46 and specimen 59 bear dense and short setae over the entire body surface that are orientated posteriorly and are parallel to the body with their whole length, a trait associated with modern species of *Attagenus* [108]. Therefore, we conclude that these two new specimens are possibly fossil larvae of Attageninae.

#### 4.2.5. Larvae of Morphotypes with Hastisetae

**Figure 33:**
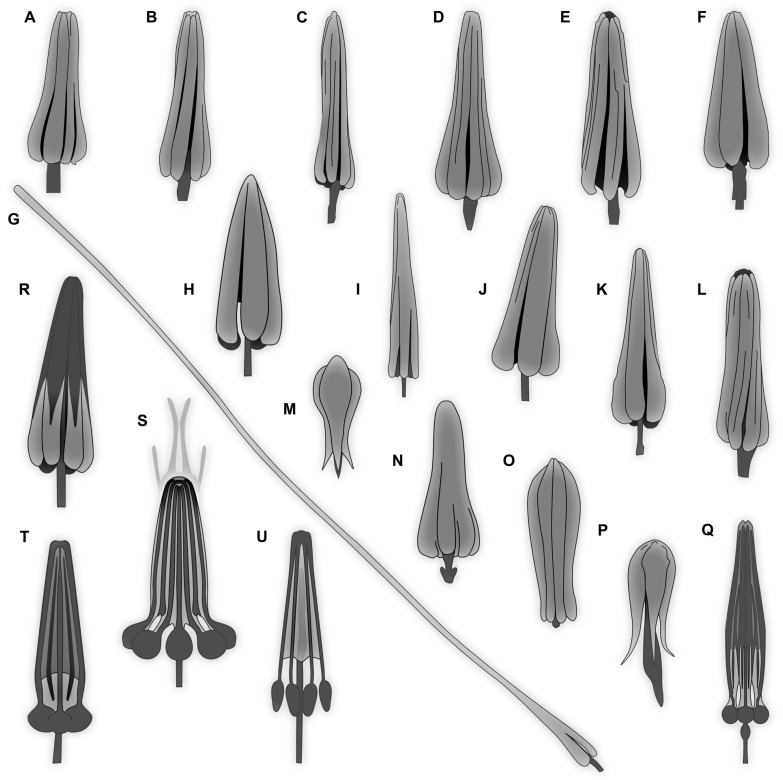
Overview of heads of hastisetae. (**A**–**Q**) Extant representatives. (**R**–**U**) Fossil representatives. (**A**) *Trogoderma granarium* Everts. (**B**) *T. glabrum* Herbst. (**C**) *T. inclusum* LeConte. (**D**) *T. angustum* Sol. (**E**) *T. versicolor* Creutz. (**F**) *T. simplex* Jayne. (**G**) *Anthrenus picturatus makolskii.* (**H**) *A. latefasciatus.* (**I**) *A. verbasci* L. (**J**) *A. olgae.* (**K**) *A. museorum* L. (**L**) *Megatoma undata* L. (**M**) *Myrmeanthrenus frontalis.* (**N**) *Cryptorhopalum triste.* (**O**) *Anthrenocerus stigmacrophilus.* (**P**) *Trinodes* sp. (**Q**) *Ctesias serra* Fabricius. (**R**) Specimen OU 33160.1 (**S**) Unidentified larva of Dermestidae, possibly Megatominae. (**T**) New specimen PED 4043. (**U**) New specimen PED 3961. (**A**–**F**,**I**,**K**,**L**) Redrawn after Elbert [9]. (**G**,**H**,**J**) Redrawn after Ruzzier et al. [8]. (**M**–**P**) Redrawn after Kiselyova and McHugh [26]. (**Q**) Redrawn after Kadej [27]. (**R**) Redrawn after a new image of already published specimen from Schmidt et al. [43]. (**S**) Redrawn after Poinar and Poinar [10].

**Figure 34:**
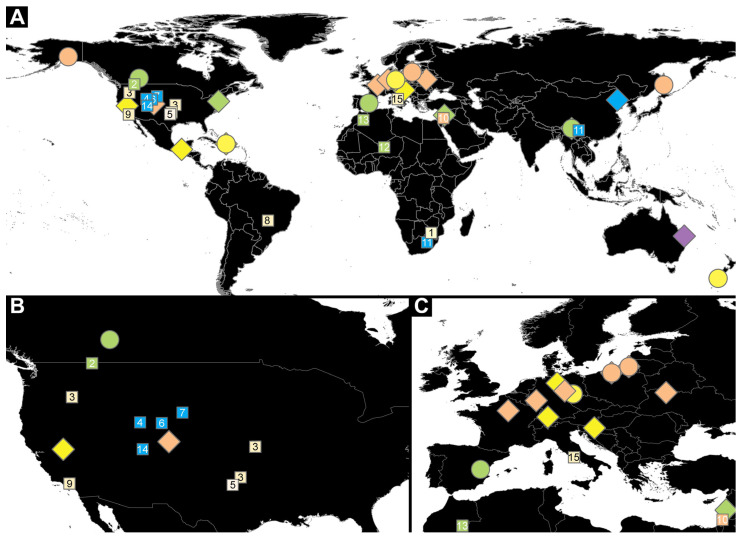
Occurrence of Dermestidae in the fossil record (adults and larvae), including traces (burrowing activity in fossilized bones), depending on their geological locality (imaged on a map provided by QGIS, licensed CC-0 1.0 Public Domain). (**A**) Occurrence of Dermestidae all over the world with adults (diamonds), juveniles (circles), and traces (numbered squares). (**B**) Occurrence of Dermestidae in the USA. (**C**) Occurrence of Dermestidae in Europe, North Africa, and Middle East. Color-code follows the geological time scale color-code: Triassic = violet; Jurassic = blue; Cretaceous = green; Eocene = orange; Miocene = yellow; Pleistocene = beige. Literature sources of fossilized traces: Kitching [44] (square 1); Rogers [45] (square 2); Martin & West [46] (square 3); Hasiotis et al. [47] (square 4); West & Hasiotis [48] (square 5); Britt et al. [49] (square 6); Bader et al. [50] (square 7); Dominato et al. [51] (square 8); Holden et al. [52] (square 9); Huchet et al. [53] (square 10); Xing et al. [54] (square 11); Höpner & Bertling [55] (square 12); Shears-Ozeki [56] (square 13); McHugh et al. [57] (square 14); Gatta et al. [58] (square 15).

Among extant larvae of Dermestidae, hastisetae are characteristic of Megatominae (Figure 33), with the exception of the larvae of Trinodini within Trinodinae [110,113]. Therefore, we identify all of the new specimens with hastisetae as fossil larvae of Megatominae. Notably, Megatominae is an ingroup of Dermestidae with very diverse representatives [12], with high ecological impact, which is likely linked to the presence of hastisetae in the group [20,114]. The substantial number of reported fossil larvae of Megatominae [10,31,42,43,97,98,101,103,104], as well as the 30 new specimens described here (potentially representatives of Megatominae or of a closely related extinct group), suggests that the representatives of this group were broadly distributed in ancient ecosystems. This is further emphasized by the fact that the fossils reported here originate from different geological periods and geographical areas of the past (Figure 34).

### 4.3. Possible Identities of Specimens of Megatominae

Among the newly identified specimens exhibiting hastisetae, there are notable differences in morphology. Some larvae display hastisetae in big tufts exclusively on their posterior abdomen, whereas others possess them distributed across the entire body in bands transverse to the body axis or in small groups laterally. Although the presence of hastisetae implies these samples to be representatives of Megatominae, the state of preservation in some specimens as well as the syn-inclusions within amber do not allow for more detailed identifications.

#### 4.3.1. Larvae of Morphotype 5

Firstly, larvae of this morphotype have an elongated body, with each segment of the trunk bearing hastisetae. Secondly, the hastisetae are organized into small cushion-like areas on the tergites, pointing from the lateral sides of the tergites towards the center of the tergites, a pattern resembling that of some larvae of *Trogoderma* and *Cryptorhopalum* (their figure 1) [26]. Additionally, at the posterior end of the trunk, a tuft of hastisetae is present on each lateral surface of the tergite, with the heads of the hastisetae pointing medially. A longer posterior brush of setae at the trunk end is also observed. The overall habitus of the new larva closely resembles larvae of *Trogoderma* (their figure 25/4; pl. 90 p) [110,111]. Furthermore, the hastisetae of the new larvae (Figure 5C) do not appear to possess “knobs” at the posterior end of the head, making them similar to the hastiseta heads of *Trogoderma*. Even though fossil specimen 8 (OU 33160.1; Figure 6) is poorly preserved overall, several hastisetae are exceptionally well-preserved (Figure 6C–E), as previously documented by Schmidt et al. (their figure 3) [43]. Based on the morphology of the hastisetae preserved, this specimen was interpreted as being *Trogoderma*-like. Given that here we do not see the typical “knob-like” heads of hastisetae found in *Megatoma* Herbst, 1792 or *Ctesias* Stephens, 1830 (Figure 33), but instead a more “petal-like” type of hastisetae heads known from many larvae of *Trogoderma* (Figure 33), we concur with the interpretation of this specimen as *Trogoderma*-like. Two other specimens, specimens 6 and 7, that are preserved together in a single piece of amber (PED 1589; Figure 4 and Figure 5) also exhibit the same morphology described herein. Based on these morphological similarities, we conclude that these specimens are likely also larvae of Megatomini, possibly of *Trogoderma*.

#### 4.3.2. Larvae of Morphotype 6

Larvae of this morphotype exhibit a relatively spindle-shaped body with thick tufts of hastisetae on the posterior abdomen tergites. This feature is known from extant larvae of Anthrenini. In *Anthrenus*, the tufts occur from abdomen segments 5 through 7, sometimes with an additional posterior brush of hairs [108]. A significant proportion of the 30 new specimens identified as larvae of Megatominae have such a morphology. For example, specimen 5 (Specimen in Lausitz amber; Figure 2 and Figure 3), specimen 16 (SNSB BSPG 2018 III 142; Figure 9B), specimen 32 (PED 1369; Figure 12), specimen 33 (PED 3504; Figure 13), specimen 40 (PED 0809; Figure 17E), specimen 43 (PED 3892; Figure 20), specimen 44 (PED 3926; Figure 21), specimen 45 (PED 3917; Figure 22A), specimen 47 (PED 3960; Figure 23), specimen 48 (PED 3961; Figure 24), specimen 53 (PED 4051; Figure 27), and specimen 58 (PED 4379; Figure 30A). Together with other morphological traits, these specimens show a striking resemblance to the fossil larva *Anthrenus larvalis* [104]. Therefore, we propose that these fossils likely represent larvae of Anthrenini.

#### 4.3.3. Larva of Morphotype 7

Extant larvae of Ctesiini exhibit similar characteristics to larvae of Anthrenini, such as thick, long tufts of hastisetae on their posterior abdomen tergites [111]. However, in larvae of *Ctesias*, these tufts seem relatively long [111], extending from abdomen segments 3 through 7, with longer tufts on the posterior segments. Additionally, larvae of *Ctesias serra* Fabricius, 1792 are generally more elongated [115]. An example of a similar morphology with tufts of hastisetae is the newly described specimen 37 (PED 2926; Figure 16A). Unfortunately, the specimen is not accessible in dorsal view; nevertheless, the coarse tufts of posterior hastisetae are apparent and appear to begin on segment 4, a characteristic observed in extant larvae of Ctesiini (pl. 90S) [111]. The morphology of the legs, along with their resting position, appears identical to that documented for larvae of Ctesiini. Specimen 37 has longer spicisetae than those depicted in the literature on modern larvae of Ctesiini, suggesting a morphological trait not shared with extant larvae of *Ctesias*. Therefore, we suggest with caution that specimen 37 is a representative of Ctesiini.

#### 4.3.4. Larvae of Morphotype 8

Another morphotype of larvae of Megatominae observed in this study is characterized by a more elongated body form, with finer setae distributed laterally and dorsally along the trunk, and a distinct long brush of setae at the posterior end of the trunk. The hastisetae on these specimens are notably finer along the lateral regions of trunk, with some individuals displaying a sparse tuft of hastisetae posteriorly. Representatives of this morphotype include specimen 15 (SNSB BSPG 2018 III 40; Figure 9A), specimen 34 (PED 3393; Figure 14A), specimen 38 (PED 3857; Figure 16E), specimen 49 (BUB 3346; Figure 9E), and specimen 55 (PED 4168, Figure 28E). Specimen 15 exhibits tufts of hastisetae localized primarily on the posterior abdomen segments, a characteristic observed in extant larvae of *Cryptorhopalum* [70] and in ingroups of Megatomini. Additionally, lateral patches of dark setae along the tergites closely match the configuration seen in larvae of *Cryptorhopalum,* as illustrated by Kiselyova & McHugh (their figure 1) [26]. The body posture, with a distinctive backward curve, resembles that of modern larvae of Cleridae rather than the other larvae of Megatominae discussed here. Nevertheless, this body posture has also been noted for *Cryptorhopalum* larvae (their figure 1) [26]. This combination of morphological traits supports their interpretation as larva of *Cryptorhopalum*. Therefore, we suggest that specimen 15 is a larva of Megatomini.

#### 4.3.5. Larvae of Morphotype 9

Specimen 52 (PED 4043; Figure 26) also bears numerous hastisetae, though these are not restricted to the posterior abdomen, as observed in many extant larvae of Megatominae. Instead, each tergite bears laterally small tufts of fine hastisetae and robust, long spicisetae, with the spicisetae being particularly elongated on the prothorax. Additionally, the body bears short, strong, spine-like setae. The overall habitus shares several morphological similarities with larvae of *Megatoma* [116] and *Globicornis corticalis* Eichhoff, 1863 [117], both representatives of Megatomini. Notably, the antennae of larvae of *Globicornis* Latreille, 1829 (their figure 1) [117] closely resemble those of the new fossil (Figure 26D). The legs also exhibit a similar structure, and a small area located before the claw, known as the pretarsus, is clearly visible in the new specimen (Figure 26E). Based on these features, we suggest that this specimen also represents a fossil larva of Megatomini.

### 4.4. Morphological Diversity of Hairs in Fossil Larvae of Dermestidae

In Dermestidae, in addition to simple setae, known as nudisetae, we can differentiate two types of specialized setae: those with imbricate scales, called spicisetae, and those with arrow-like distal tips, called hastisetae. Spicisetae are present across most larvae of Dermestidae, but variations have been observed among different ingroups [118]. For example, spicisetae in ingroups of Dermestidae such as Dermestinae, Trinodinae, and Megatominae are typically barbed, while in some representatives of Attageninae, they are ribbed [113]. In the new fossil specimens, spicisetae also show some variability (e.g., Figure 26B), mainly in length and thickness, though the rosettes on the pedicels remain in consistent positions. Only certain larvae of Megatominae (e.g., *Trogoderma*, *Anthrenus*) and some within Trinodinae (e.g., *Trinodes*) exhibit an additional type of specialized setae, hastisetae. In total, 31 new specimens have hastisetae, therefore representing the ingroup of Megatominae in six of the amber deposits studied here. However, even in the extant larvae of Megatominae, the hastisteae are not uniform. The morphology of the heads of hastisetae often varies across different ingroups [9,26,65] and can even be a feature used for the identification of species of Dermestidae. On one hand, heads of hastisetae can have knob-like parts, as seen in the modern larvae of *Ctesias serra* (Figure 33Q); on the other hand, the heads of hastisetae can have a morphology as seen in extant larvae of *Trogoderma* (Figure 33A–F) or *Anthrenus* (Figure 33H–K). They exhibit petal-like parts with ridged microstructures separated by slots that narrow toward the tip, facilitating the entanglement of hairs from predators [119]. The morphology of the heads of hastisetae can also vary based on the body location (Figure 12C,D) [8]. Hastisetae in extant larvae also vary in other dimensions, such as pedicel length or the head width relative to length ratio (Figure 33). An extreme example is the elongated head of hastisetae in *Anthrenus picturatus makolskii* Mroczkowski, 1952 (Figure 33G after Ruzzier et al. [8]), which only has a very prolonged distal tip on membraneous areas between the tergites, but on the tergites themselves has a shorter tip [12]. Although we did not observe such extreme hastisetae in the new fossils, some clear distinctions are present (Figure 33R–U). Most hastisetae in the fossils (Figure 33R–U) resemble those of modern ingroups of Megatominae, particularly of Anthrenini, Ctesiini, and Megatomini, such as hastisetae in *Anthrenus coloratus* Reitter, 1881 and *A. vladimiri* Menier et Villemant, 1993 [27] or *Ctesias serra* (Figure 33Q) [115]. Similar hastisetae have been previously reported in Cretaceous amber [10,42]. However, the hastisetae of specimen 8 from New Zealand Miocene amber (OU 33160.1; Figure 6D,E; Figure 33R) most closely resemble those of extant larvae of *Trogoderma* (Figure 33A–F), where the proximal part of the head of hastisetae lacks visible “knob-like” structures.

### 4.5. Evolutionary Changes in Larvae of Dermestidae

Interestingly, our results indicate that larvae of Dermestidae did change over time, with extant specimens differing from Cretaceous larvae in body lengths but also with a change in the absolute length of their setae toward smaller lengths today (Figure 31). The absolute length of their hastisetae is seemingly decreasing over time as well, but this trend was revealed not to be significant (Kruskal test, *p* = 0.1857; Figure 31C). Overall, extant larvae of Dermestidae are seemingly longer, with smaller defensive setae, a signal that is also visible when looking at their relative lengths (Figure 31D–E, Supplementary Table S3).

Although a signal is visible in both of the datasets (i.e., complete dataset and dataset without fragmented specimens), these results can be interpreted differently. The investigated complete dataset probably does not cover the entire morphological diversity of larvae of Dermestidae. As seen with material from the Miocene, the sample size is limited to only six fully preserved specimens, of which two bear hastisetae (see Section 2, Supplementary Table S2, and Figure 31E). Additionally, representation of the data using violin plots, a type of plot that pictures densities of points, was not possible when investigating the relative length of hastisetae ratio because densities were represented by simple lines. This was likely due to a small sampling size, again not covering the whole morphological variability. Therefore, these results were represented with box plots. For the longest setae, it seems that the data are not continuous for specimens from the Eocene and Miocene epochs due to a limited sample size (Figure 31B). For the Eocene, a single specimen has setae of more than 3 mm in length (specimen 15); for the Miocene this is more extreme, with a single specimen having setae of about 5 mm (specimen 7) and one with extremely small setae of less than 0.1 mm (specimen 9).

The available material regarding larvae of Dermestidae that can be used for analyses is very limited, for both extant and fossil larvae, limiting the use of powerful analyses and restricting us to the non-parametric Kruskal test and post hoc Dunn tests. The results on the whole group of Dermestidae potentially represent differences between groups (i.e., interspecific variation) and not a whole-group trend. As seen with the dataset investigating only representatives of Megatominae, the differences in body length over time are actually non-significant (Figure 32A; Supplementary Table S3); thus, the intergroup bias has probably been removed. The complete dataset on the entire group of Dermestidae is likely affected by the presence of additional ingroups of Dermestidae in the Cretaceous that are not represented in the extant specimens. Extant specimens that were investigated are mainly representatives of *Anthrenus* and, in general, Megatominae. Interestingly, specimens from the Cretaceous had a more diverse group coverage, including fossils of Megatominae, Attegeninae, Trinodinae, and Dermestinae. Yet, this signal is still present when investigating only the group of Megatominae (Figure 32B,D,E). Extant larvae are showing smaller setae as well as smaller setae- and hastisetae-to-body ratios.

With the 36 new fossil specimens of (possible) larvae of Dermestidae reported here, we have greatly improved the available fossil record for further studies. However, to advance our understanding of the diversity and morphology of Dermestidae in deep time, further research incorporating a larger number of extant specimens and new fossil specimens is required, particularly from the Miocene and Eocene, and from groups other than Megatominae. Ideally, these fossils should include fully preserved specimens to enable a comprehensive study of their morphology. Investigating larval forms is especially relevant to investigate evolution processes at work [120,121,122,123].

Despite the findings, certain biases need to be considered here. The literature on extant larvae is often focused on late- or last-stage larvae. Amber tends to preserve smaller, often also meaning earlier-stage, larvae. Hence, the difference between amber and the extant fauna could be interpreted as a mere sampling difference, with earlier-stage larvae in the fossil and later-stage larvae in the extant fauna. Yet, as we have ambers from different time periods, we can also see that a signal is apparent here; if amber was indeed preserving smaller specimens, a signal should be visible between Eocene/Miocene larvae and modern ones. As the sampling biases are similar for the different ambers, it is unlikely that this signal is influenced by sampling biases. It therefore seems likely that we have a true signal here. In addition, the fact that there are no significant differences in the body lengths of larvae in Megatominae over time shows that the impact of entrapment size is too low to affect the true signal.

So far, fossil beetle larvae were largely in the morphological range well known of modern forms concerning quantitative aspects. An exception is represented by larvae of Scraptiidae, which had larger terminal ends in the past [124,125], but here a possible sampling bias of early versus later stages (early in the fossil record, late in the extant fauna) could potentially explain the difference. In larvae of other holometabolans closely related to beetles, such as snakeflies and lacewings, clear losses of quantitative morphological variation could be recognized [126,127]. Dermestidae, and precisely the ingroup Megatominae, is potentially the first ingroup of Coleoptera for which we now have a comparable pattern, with more quantitative variation in the Cretaceous than in the modern fauna.

### 4.6. Defensive Setae in Representatives of Euarthropoda

The evolution of defensive setae in Euarthropoda has happened at least four times, in Arachnida, Lepidoptera, Polyxenida, and Dermestidae [114]. This atypical mechanical defense (i.e., modified defensive setae for entangling) [119] is not restricted to some larvae of Dermestidae; it is also found in the group of the bristly millipedes (Polyxenida) [8]. Bristly millipedes and other millipedes are usually detritivores and are largely preyed on by ants and isopodan crustaceans [128]. They have developed multiple sets of defense mechanisms, one of the most common being chemical compounds, produced by the defensive glands (i.e., ozadenes or repugnatory glands) [129,130]. Bristly millipedes differ in many aspects from their other diplopodan relatives. Defense-wise, they rely on mechanical defense in the form of an intricate set of “barbate trichomes” (although these structures are not trichomes, but setae, likely a historical use of the French term “trichome” meaning “modified setae” remained; e.g., setae, Pocock [131]; e.g., trichomes, Brölemann [132]). Although representatives of Dermestidae and Polyxenidae are not closely related, it is not unusual to mistake one for the other. We can therefore expect that at least some larvae of Dermestidae in collections may be erroneously identified as bristly millipedes, or the other way around.

Due to the high morphological similarities between the two groups (for comparison, see Figure 1A,B,F,G, Figure 10A–D and Figure 11A,B,E,F), we expect very similar functions of their modified setae (i.e., trichomes in Polyxenida and hastisetae/spicisetae in Dermestidae). Physical defense by modified setae has been revealed to be quite effective in entangling predators [8,13,119]. However, some predators have developed ways to circumvent this “hairy” defense [8,13], for example, ants, the common predators of bristly millipedes. Ants of the group *Thaumatomyrmex* Mayr, 1887 have been seen stripping down representatives of Polyxenida, removing their hairy tufts until the body is accessible [133,134]. In the parasitoid wasps of *Laelius* Ashmead, 1893, representatives avoid the entangling defense of larvae of Dermestidae, likely as a function of their small size (e.g., on larvae of *Trogoderma*) [135]. It is very likely that the minute size of these parasitoid wasps allows them to access their prey without triggering detachable hastisetae [136].

Despite many similarities between skin, larder, or carpet beetle larvae and bristly millipedes, their defensive behaviors and mechanical release of setae against predators are very different. Larvae of Dermestidae have a more active response: they curl their body toward the predator and orient their tergites in order to increase the amount of hastisetae detached [8,13]. Additionally, the specimens of Dermestidae with posteriorly and dorsally oriented brushes of long hastisetae can actively raise the hairs, providing an even larger shielded area around them in times of danger [137]. Bristly millipedes can actively spread their terminal setae-bundles like “fans” [138] as well, but will additionally move them in the direction of the predator, in order to deter it from attacking [138]. Further differences include the hastisetae of Dermestidae breaking along the pedicel, something that is not found in bristly millipedes, where the entire setae are released [8].

Lepidopteran caterpillars have also evolved hairs that are used for defense, although they are structurally different as they are not barbed like the hairs of larvae of Dermestidae or Polyxenida. The hairs of lepidopteran caterpillars can serve different functions, including physical defense [139,140], chemical defense [141,142,143], and behavioral defense [144,145]. These hairs are both effective against vertebrate predators such as birds and against non-vertebrate predators such as ants or beetles [139,146], as well as in some cases also parasitoids [140]. Caterpillars’ use of hairs in physical defense is largely to prevent predators making contact with the larva, or to increase the handling difficulty [139,147], which means that the length of the hairs is especially important. This is often combined with aposematic coloring, to prevent the caterpillar from being attacked in the first place [148]. Chemical defense is performed via glandular setae either releasing toxins [149,150] or sometimes other deterring chemicals [142,143]. The kind of structurally modified setae necessary for chemical defense have been found in a fossil caterpillar in Eocene Baltic amber [151]. These glandular setae are visually different to those of Dermestidae or Polyxenidae. Instead of “arrowhead-like” structures, they have a “swollen” tip that holds a droplet of the chemical deterrent [142,151]. Behavioral defense is most often employed in conjunction with the previously mentioned types of defenses. Examples of this kind of defensive behavior are curling up or swaying movements [146]. Typically, these behaviors are used to further lower the risk of successful predation, by protecting more vulnerable areas of the body or more prominently displaying these hairs as a way of visually signifying inedibility. Except for the species using glandular hairs for chemical defense, lepidopteran larvae do not show the high degree of specialization in their hairs that is found in larvae of Dermestidae.

Concerning the fossil record, the pattern in larvae of Dermestidae is different from that observed in lepidopteran caterpillars. Lepidopteran caterpillars start out with rather short defensive setae in the Cretaceous (pers. obs. JTH), and the maximum relative lengths become progressively longer over time, reaching modern conditions, with a wide range from very short to very long defensive setae most likely in the Miocene. In larvae of Dermestidae, the pattern is apparently reversed, with a wide variation in relative lengths of defensive setae in the Cretaceous and only rather short defensive setae in the modern fauna.

### 4.7. Temporal and Spatial Occurrences of Larvae of Dermestidae

As the new material demonstrates, larvae of Dermestidae are not only omnipresent in modern ecosystems; they have been significantly contributing to ecological cycles with their feeding activities since at least the Cretaceous. We have summarized various localities where fossil representatives in the form of both larvae and adults have been found (Figure 34: circles, diamonds). In addition, possible trace fossils of the burrowing activities of fossil larvae of Dermestidae increase the spatial occurrence of this group in the past (Figure 34: squares; for literature sources of ichnofossils see figure caption). Seven different geological localities are represented by new fossil larvae from this publication, expanding the spatial coverage of the group to encompass regions from the tropics to warm temperate settings at the time of deposit formation. The larvae preserved in Kachin Myanmar amber show especially high morphological diversity. The diversity of the forms summarized here indicates that larvae of Dermestidae had evolved differentiated ecological roles as early as the Cretaceous period. Interestingly, Kachin Myanmar amber provided the oldest inclusions of larvae of Dermestinae. We were also able to identify representatives of four or possibly five (Dermestinae, Megatominae, Attageninae, Trinodinae, and possibly Orphilinae) of six major modern ingroups of Dermestidae in the deposit. The larva of Dermestinae from this publication is the oldest known larva of this ingroup, and yet it already exhibited rather modern-looking morphology.

Our extensive study of fossilized larvae of Dermestidae preserved in amber suggests that their rarity in the scientific literature does not reflect their actual occurrence. Instead, this perceived scarcity likely arises from a historical lack of focus on the immature stages of beetles in paleontological research. Additionally, this effect may be compounded by the potential underrepresentation of fossil larvae of Dermestidae in museum collections. Nonetheless, the availability of such larvae at numerous amber traders emphasizes that these inclusions were more frequently preserved in amber than previously documented (see Supplementary Figure S1 and S2). Hopefully, this availability of material will continue to expand our knowledge of past diversity and ecology within Dermestidae.

## 5. Conclusions

In this study, we did not only expand the fossil record of Dermestidae larvae and show that they were more abundant in ancient ecosystems than previously assumed, but we also quantified and compared the morphological diversity of larvae of Dermestidae through time. Of the 36 new specimens, 35 were clearly identified as Dermestidae, with most of them being representatives of Megatominae. Nevertheless, we also identified representatives of other ingroups of Dermestidae, such as Trinodinae, Attageninae, and Dermestinae. The diversity of morphotypes was especially expressed in the specimens from Kachin amber. A single specimen from the Miocene is possibly not a larva of Dermestidae but might still be a representative of Bostrichoidea (wider group including Dermestidae). However, a clear identification remained difficult due to partially concealed mouthparts. The analyses of these new specimens, in regard to their body length and setae, with other fossil specimens from the literature and also extant specimens revealed significant differences in morphology between the Cretaceous and extant larvae. This was true for both the complete dataset and for the subdataset of only larvae of Megatominae. Cretaceous larvae of Dermestidae had longer setae than extant ones. Both nudisetae and spicisetae are significantly longer in the extinct specimens from the Cretaceous, but the length of the hastisetae did not change significantly. The sample size of our dataset is relatively small, but these results still point towards a trend of decreased setae length. Even though we showed that there were differences in the defensive setae between the fossil and extant larvae, we also showed that many of the Cretaceous forms already closely resembled their modern forms. This possibly indicates that the fossil larvae of Dermestidae fulfilled similar ecological roles to their modern counterparts. However, our results do not allow conclusions regarding evolutionary pressures that might have driven the morphological changes between fossil and extant representatives of Dermestidae.

## Data Availability

The original contributions presented in the study are included in the article/Supplementary Materials, further inquiries can be directed to the corresponding author.

## Supplementary Materials

The following supporting information can be downloaded at https://www.mdpi.com/article/10.3390/insects16070710/s1, Figure S1: Morphological diversity of fossil larvae of Dermestidae in Baltic amber visible at the ambertreas-ure4u.com internet site; Figure S2: Morphological diversity of fossil larvae of Dermestidae in Baltic amber visible at the ambertreasure4u.com internet site; Figure S3: Volume renderings of head in ventral view (A) and in dorsal view (B) based on synchrotron X-ray tomography, a raw scan of synchrotron X-ray tomography (C), and volume renderings of the maxillary endites’ area in different views (D–H); Table S1: Information on the fossil and extant larvae of Dermestidae studied in this publication, including new (marked with darker shades of yellow, orange and green) and already published specimens (marked with paler colors); Table S2: Measurements of the new and already published fossil and extant larvae of Dermestidae; Table S3: Results of the analyses on the complete dataset, dataset without fragmentary specimens, and dataset of only larvae of Megatominae.
