# Supplementary material for: New Amber Fossils Indicate That Larvae of Dermestidae Had Longer Defensive Structures in the Past
Source: Insects. 2025 Jul 10;16(7):710. doi: 10.3390/insects16070710 (PMC12295104; doi:10.3390/insects16070710)
Supplement: Supplementary file 1 [file insects-16-00710-s001.zip › Supplementary Table S1.pdf]

Specimen nr.	Source	Figure nr.	Amber	Age	Accession nr.	ID
1	Kiselyova & McHugh 2006 [26]	fig. 1A–C	Dominican	Early-Mid Miocene	NA	*Cryptorhopalum (?)*
2	Poinar & Háva 2015 [33]	fig. 21	Dominican	Early-Mid Miocene	C-7-7A	*Apsectus*
3	Poinar & Háva 2015 [33]	fig. 22	Dominican	Early-Mid Miocene	C-7-7B	*Apsectus*
4	Háva 2023 [31]	figs. 10–11	Dominican	Early-Mid Miocene	JHAC	*Cryptorhopalum*
5	Zippel 2023, thesis [72]; HERE	fig. 2G; Figs 2–3	Lausitz	Miocene	NO NUMBER	Megatominae
6	NEW SPECIMEN	Fig. 4	Dominican	Early-Mid Miocene	PED 1589	Megatominae
7	NEW SPECIMEN	Figs 4-5	Dominican	Early-Mid Miocene	PED 1589	Megatominae
8	Schmidt et al. 2018 [43]; HERE	fig. 3I ; Fig. 6	Roxburgh	Early Miocene	OU 33160.1	*Trogoderma (?)*
9	NEW SPECIMEN	Figs 7–8	Hyde	Miocene	OU 33636.3	Orphilinae?
10	Háva et al. 2006 [35]	fig. 11	Baltic	Eocene	GPIH 4466/JHAC JDC9692	*Trogoderma larvalis*
11	Kadej & Háva 2011 [65]	figs. 1–4	Baltic	Eocene	5369	*Trinodes*
12	Háva 2022a [97]	fig. 3	Baltic	Eocene	NBSD 6069	*Anthrenus*
13	Grimaldi et al. 2018 [98]	figs. 8–14F	Alaskan	Eocene	AMNH LC-II-B4	Megatominae
14	Perkovsky et al. 2021 [60]	figs. 1–8	Sakhalinnian	Eocene	3387-1060	*Trogoderma ainu*
15	NEW SPECIMEN	Fig. 9	Baltic	Eocene	SNSB BSPG 2018 III 40	Megatominae
16	NEW SPECIMEN	Fig. 9	Baltic	Eocene	SNSB BSPG 2018 III 142	Megatominae
17	Poinar & Poinar 2016 [10]	figs. 1–2	Myanmar	Cretaceous	NO NUMBER	Dermestoidea
17	Rasnitsyn et al. 2017 [99]	fig. 3 J–L	Myanmar	Cretaceous	NO NUMBER	Dermestoidea
18	Peñalver et al. 2017 [42]	fig. 6	Myanmar	Cretaceous	AMNH Bu-SA5	Megatominae
19	Zhang 2017 [100]	p 441	Myanmar	Cretaceous	NO NUMBER	NA
20	Poinar 2019 [101]	fig. 29	Myanmar	Cretaceous	NO NUMBER	NA
20	Poinar 2010 [102]	fig. 15	Myanmar	Cretaceous	NO NUMBER	NA
21	Peris & Rust 2020 [103]	fig. 4B	Myanmar	Cretaceous	NO NUMBER	NA
22	Háva 2022b [104]	fig. 1	Myanmar	Cretaceous	In. 19107-16	*Anthrenus larvalis*
22	Ross & York 2000 [105]		Myanmar	Cretaceous	NO NUMBER	*Anthrenus larvalis*
22	Cockerell 1917 [64]		Myanmar	Cretaceous	NO NUMBER	*Dermestes larvalis*
23	Háva 2023 [31]	fig. 9	Myanmar	Cretaceous	JH/TR/1	*Trogoderma*
24	Peñalver et al. 2023 [106]	fig. 1	Spanish	Cretaceous	SJNB2012-31-01	*Orphilus*
25	Peñalver et al. 2023 [106]	fig. 3	Spanish	Cretaceous	SJNB2012-11	*Orphilus (?)*
26	Peñalver et al. 2023 [106]	suppl.fig.	Spanish	Cretaceous	ES-07-39	*Orphilus (?)*
27	Peñalver et al. 2023 [106]	suppl.fig.	Spanish	Cretaceous	MCNA 12063	*Orphilus (?)*
28	NEW SPECIMEN	Fig. 10	Canadian	Cretaceous	TMP 96.9.366	Dermestidae
29	NEW SPECIMEN	Fig. 10	Canadian	Cretaceous	TMP 96.9.393a	Dermestidae
30	NEW SPECIMEN	Fig. 10	Canadian	Cretaceous	TMP 96.9.393b	Dermestidae
31	NEW SPECIMEN	Fig. 11	Myanmar	Cretaceous	PED 2550	Megatominae
32	Haug et al. 2024 [73]; HERE	fig. 8F; Fig. 12	Myanmar	Cretaceous	PED 1369	Megatominae
33	NEW SPECIMEN	Fig. 13	Myanmar	Cretaceous	PED 3504	Megatominae
34	NEW SPECIMEN	Fig. 14	Myanmar	Cretaceous	PED 3393	Megatominae
35	NEW SPECIMEN	Fig. 14	Myanmar	Cretaceous	PED 2929	Megatominae
36	NEW SPECIMEN	Fig. 15	Myanmar	Cretaceous	PED 3663	Dermestinae
37	NEW SPECIMEN	Fig. 16	Myanmar	Cretaceous	PED 2926	Megatominae
38	NEW SPECIMEN	Fig. 16	Myanmar	Cretaceous	PED 3857	Megatominae
39	NEW SPECIMEN	Fig. 17	Myanmar	Cretaceous	PED 0707	Megatominae
40	NEW SPECIMEN	Fig. 17	Myanmar	Cretaceous	PED 0809	Megatominae
41	NEW SPECIMEN	Fig. 18	Myanmar	Cretaceous	PED 0647	Megatominae
42	NEW SPECIMEN	Fig. 19	Myanmar	Cretaceous	PED 1849	Trinodinae
43	NEW SPECIMEN	Fig. 20	Myanmar	Cretaceous	PED 3892	Megatominae
44	NEW SPECIMEN	Fig. 21	Myanmar	Cretaceous	PED 3926	Megatominae
45	NEW SPECIMEN	Fig. 22	Myanmar	Cretaceous	PED 3917	Megatominae
46	NEW SPECIMEN	Fig. 22	Myanmar	Cretaceous	PED 3705	Attageninae
47	NEW SPECIMEN	Fig. 23	Myanmar	Cretaceous	PED 3960	Megatominae
48	NEW SPECIMEN	Fig. 24	Myanmar	Cretaceous	PED 3961	Megatominae
49	NEW SPECIMEN	Fig. 9	Myanmar	Cretaceous	BUB3346	Megatominae
50	NEW SPECIMEN	Fig. 25	Myanmar	Cretaceous	BUB3184	Megatominae
51	NEW SPECIMEN	Fig. 25	Myanmar	Cretaceous	BUB3353	Megatominae
52	NEW SPECIMEN	Fig. 26	Myanmar	Cretaceous	PED 4043	Megatominae
53	NEW SPECIMEN	Fig. 27	Myanmar	Cretaceous	PED 4051	Megatominae
54	NEW SPECIMEN	Fig. 28	Myanmar	Cretaceous	PED 4148	Megatominae
55	NEW SPECIMEN	Fig. 28	Myanmar	Cretaceous	PED 4168	Megatominae
56	NEW SPECIMEN	Fig. 29	Myanmar	Cretaceous	PED 4406	Megatominae
57	NEW SPECIMEN	Fig. 29	Myanmar	Cretaceous	PED 4409	Megatominae
58	NEW SPECIMEN	Fig. 30	Myanmar	Cretaceous	PED 4379	Megatominae
59	NEW SPECIMEN	Fig. 30	Myanmar	Cretaceous	PED 4380	Attageninae
ex01	Kadej et al. 2013a [66]	/	NA	Extant	NA	*Anthrenus*
ex02	Kadej et al. 2013a [66]	/	NA	Extant	NA	*Anthrenus*
ex03	Kadej et al. 2013a [66]	/	NA	Extant	NA	*Anthrenus*
ex04	Kadej et al. 2013b [67]	/	NA	Extant	NA	*Anthrenus*
ex05	Kadej & Guziak 2017 [69]	figs. 1–2	NA	Extant	NA	Attageninae
ex06	Kadej et al. 2017 [68]	figs. 1–3	NA	Extant	NA	*Trogodermina*
ex07	Kadej & Guziak 2017 [69]	/	NA	Extant	NA	Megatominae
ex08	Kiselyova 2008 [71]	fig. 1	NA	Extant	NA	Megatominae?
ex09	Kiselyova 2008 [71]	fig. 11	NA	Extant	NA	Megatominae
ex10	Kiselyova 2008 [71]	fig. 20	NA	Extant	NA	Megatominae
ex11	Kiselyova 2002 [70]	fig. 1	NA	Extant	NA	*Cryptorhopalum*
