# Supplementary material for: New Amber Fossils Indicate That Larvae of Dermestidae Had Longer Defensive Structures in the Past
Source: Insects. 2025 Jul 10;16(7):710. doi: 10.3390/insects16070710 (PMC12295104; doi:10.3390/insects16070710)
Supplement: Supplementary file 1 [file insects-16-00710-s001.zip › Supplementary Table S2.pdf]

| Specimen nr. | Accession nr. | Preservation | Body length (mm) | Longest seta (mm) | Longest hastiseta (mm) | Seta/Body ratio | Hastiseta/Body ratio |
|---|---|---|---|---|---|---|---|
| 1 | NA | full | NA | NA | NA | NA | NA |
| 2 | C-7-7A | full | 0.88 | 0.9247 | NA | 1.0507954545 | NA |
| 3 | C-7-7B | full | 0.88 | 1.057 | NA | 1.2011363636 | NA |
| 4 | JHAC | full | 5.2 | NA | NA | NA | NA |
| 5 | NA | full | 1.8 | 1.1 | 0.3 | 0.6111111111 | 0.1666666667 |
| 6 | PED 1589 | fragm | 4.19 | 3.87 | 1.41 | 0.923627685 | 0.3365155131 |
| 7 | PED 1589 | full | 8.43 | 4.97 | 1.4 | 0.5895610913 | 0.1660735469 |
| 8 | OU 33160.1 | hasti | NA | NA | 0.25 | NA | NA |
| 9 | OU 33636.3 | full | 2.04 | 0.1 | NA | 0.0490196078 | NA |
| 10 | GPIH 4466 | full | 3.8 | 0.53 | 1.23 | 0.1394736842 | 0.3236842105 |
| 11 | 5369 | full | 1 | 0.423 | NA | 0.423 | NA |
| 12 | NBSD 6069 | full | NA | NA | NA | NA | NA |
| 13 | AMNH LC-II-B4 | fragm | NA | 0.4 | 0.21 | NA | NA |
| 14 | 3387-1060 | full | 1 | 0.91 | 0.23 | 0.91 | 0.23 |
| 15 | SNSB BSPG 2018 III 40 | full | 2.53 | 3.16 | NA | 1.2490118577 | NA |
| 16 | SNSB BSPG 2018 III 142 | full | 1.35 | 0.54 | 0.21 | 0.4 | 0.1555555556 |
| 17 | NO NUMBER | hasti | NA | NA | 0.33 | NA | NA |
| 17 | NO NUMBER | hasti | NA | NA | NA | NA | NA |
| 18 | AMNH Bu-SA5 | hasti | NA | NA | 0.38 | NA | NA |
| 19 | NA | full | NA | NA | NA | NA | NA |
| 20 | NA | full | 2.37 | 1.24 | NA | 0.5232067511 | NA |
| 20 | NA | full | NA | NA | NA | NA | NA |
| 21 | NA | full | 1.67 | 2.547 | 0.713 | 1.5251497006 | 0.4269461078 |
| 22 | In. 19108 | full | NA | NA | NA | NA | NA |
| 22 | In. 19108 | full | 0.75 | 3.32 | 0.41 | 4.4266666667 | 0.5466666667 |
| 22 | In. 19108 | full | NA | NA | NA | NA | NA |
| 23 | NA | fragm | 2 | NA | NA | NA | NA |
| 24 | SJNB2012-11, ES-07-39 | fragm | NA | NA | NA | NA | NA |
| 25 | NA | full | 0.83 | NA | NA | NA | NA |
| 26 | NA | fragm | NA | NA | NA | NA | NA |
| 27 | NA | fragm | NA | NA | NA | NA | NA |
| 28 | TMP 96.9.366 | fragm | 2 | 1.25 | NA | 0.625 | NA |
| 29 | TMP 96.9.393a | hasti | NA | 1.2 | NA | NA | NA |
| 30 | TMP 96.9.393b | hasti | NA | NA | NA | NA | NA |
| 31 | PED 2550 | full | 2.78 | 3.82 | 0.66 | 1.3741007194 | 0.2374100719 |
| 32 | PED 1369 | full | 1.74 | 3.41 | 0.72 | 1.9597701149 | 0.4137931034 |
| 33 | PED 3504 | full | 1.55 | 2.96 | 0.56 | 1.9096774194 | 0.3612903226 |
| 34 | PED 3393 | full | 2.41 | NA | 0.27 | NA | 0.112033195 |
| 35 | PED 2929 | full | 1.27 | 3.25 | 0.53 | 2.5590551181 | 0.4173228346 |
| 36 | PED 3663 | full | 4.59 | 1.33 | NA | 0.2897603486 | NA |
| 37 | PED 2926 | full | 1.63 | 1.64 | 0.65 | 1.0061349693 | 0.3987730061 |
| 38 | PED 3857 | full | 1.98 | 3.43 | 0.71 | 1.7323232323 | 0.3585858586 |
| 39 | PED 0707 | fragm | 2.99 | 1.73 | 0.46 | 0.5785953177 | 0.1538461538 |
| 40 | PED 0809 | fragm | 3.73 | 4.57 | 0.47 | 1.2252010724 | 0.1260053619 |
| 41 | PED 0647 | full | 1.99 | 0.9 | 0.41 | 0.4522613065 | 0.2060301508 |
| 42 | PED 1849 | full | 1.6 | 0.37 | NA | 0.23125 | NA |
| 43 | PED 3892 | full | 0.77 | 1.63 | 0.41 | 2.1168831169 | 0.5324675325 |
| 44 | PED 3926 | full | 1.1 | 4.76 | 0.76 | 4.3272727273 | 0.6909090909 |
| 45 | PED 3917 | full | 0.98 | 2.78 | 0.52 | 2.8367346939 | 0.5306122449 |
| 46 | PED 3705 | full | 2.7 | 2.17 | NA | 0.8037037037 | NA |
| 47 | PED 3960 | full | 1.75 | 3.9 | 0.5 | 2.2285714286 | 0.2857142857 |
| 48 | PED 3961 | full | 0.6 | 2.25 | 0.35 | 3.75 | 0.5833333333 |
| 49 | BUB3346 | full | 1.2 | 1.2 | 0.25 | 1 | 0.2083333333 |
| 50 | BUB3184 | full | 2.9 | 2.2 | 1.2 | 0.7586206897 | 0.4137931034 |
| 51 | BUB3353 | full | 3.6 | 6.75 | 1.6 | 1.875 | 0.4444444444 |
| 52 | PED 4043 | full | 3.75 | 3 | 0.65 | 0.8 | 0.1733333333 |
| 53 | PED 4051 | full | 3.6 | 5 | 2.05 | 1.3888888889 | 0.5694444444 |
| 54 | PED 4148 | full | 3.6 | 5.51 | 1 | 1.5305555556 | 0.2777777778 |
| 55 | PED 4168 | full | 1.35 | 1.9 | 0.3 | 1.4074074074 | 0.2222222222 |
| 56 | PED 4406 | full | 4.2 | 1.65 | 0.6 | 0.3928571429 | 0.1428571429 |
| 57 | PED 4409 | fragm | 5.38 | 3.11 | 1.18 | 0.5780669145 | 0.219330855 |
| 58 | PED 4379 | full | 0.9 | 2.5 | 0.3 | 2.777777777 | 0.33333333 |
| 59 | PED 4380 | full | 2.1 | 1.5 | NA | 0.7142857143 | NA |
| ex01 | NA | full | 1.4 | 0.27 | 0.17 | 0.192857143 | 0.1214285714 |
| ex02 | NA | full | 1.75 | 0.52 | 0.2 | 0.297142857 | 0.1142857143 |
| ex03 | NA | full | 1.8 | 0.36 | 0.41 | 0.2 | 0.2277777778 |
| ex04 | NA | full | 2.1 | 0.57 | 0.11 | 0.27142857 | 0.052380952 |
| ex05 | NA | full | 10 | 0.254 | NA | 0.0254 | NA |
| ex06 | NA | full | 5.75 | 1.49 | 0.5 | 0.259130435 | 0.086956522 |
| ex07 | NA | full | 7.75 | 0.254 | 0.46 | 0.032774194 | 0.059354839 |
| ex08 | NA | full | 3.76 | 0.31 | NA | 0.082446809 | NA |
| ex09 | NA | full | 4.33 | 0.37 | NA | 0.085450346 | NA |
| ex10 | NA | full | 3.91 | 1.61 | 0.33 | 0.411764706 | 0.084398977 |
| ex11 | NA | full | 3.8 | 2.4 | 0.86 | 0.631578947 | 0.226315789 |
