# Supplementary material for: New Amber Fossils Indicate That Larvae of Dermestidae Had Longer Defensive Structures in the Past
Source: Insects. 2025 Jul 10;16(7):710. doi: 10.3390/insects16070710 (PMC12295104; doi:10.3390/insects16070710)
Supplement: Supplementary file 1 [file insects-16-00710-s001.zip › Supplementary Table S3.pdf]

**Full dataset**		
Body length	Kruskal	0.05561
Longest seta length	**Kruskal**	**0.0001**
Dunn test	uncorrected	corrected
Extant:Miocene	0.1056	0.4225
Extant:Eocene	0.5955	0.5955
**Extant:Cretaceous**	**3.61e-05**	**0.0002**
Miocene:Eocene	0.3392	0.6783
Miocene:Cretaceous	0.1645	0.4936
**Eocene:Cretaceous**	**0.0085**	**0.0423**
Longest hastiseta length	Kruskal	0.09
Setae/Body ratio	**Kruskal**	**2.87e-05**
Dunn test	uncorrected	corrected
Extant:Miocene	0.0585	0.2693
Extant:Eocene	0.1669	0.3339
**Extant:Cretaceous**	**2.18e-06**	**1.31e-05**
Miocene:Eocene	0.7003	0.7003
Miocene:Cretaceous	0.1282	0.3847
Eocene:Cretaceous	0.0585	0.234
Hastisetae/body ratio	**Kruskal**	**0.0028**
Dunn test	uncorrected	corrected
Extant:Miocene	0.2373	0.9492
Extant:Eocene	0.1654	0.8268
**Extant:Cretaceous**	**0.0002**	**0.0013**
Miocene:Eocene	0.8647	0.8647
Miocene:Cretaceous	0.2594	0.7781
Eocene:Cretaceous	0.3685	0.737

**Without fragmented specimens**		
Body length	**Kruskal**	**0.0394**
Dunn test	uncorrected	corrected
Extant:Miocene	0.1592	0.6367
Extant:Eocene	**0.0487**	0.2434
**Extant:Cretaceous**	**0.0052**	**0.0312**
Miocene:Eocene	0.5647	1
Miocene:Cretaceous	0.5451	1
Eocene:Cretaceous	0.8716	0.8716
Longest seta length	**Kruskal**	**0.0002**
Dunn test	uncorrected	corrected
Extant:Miocene	0.3183	0.955
Extant:Eocene	0.5046	1
**Extant:Cretaceous**	**4.36e-05**	**0.0003**
Miocene:Eocene	0.7779	0.7779
Miocene:Cretaceous	0.0646	0.2418
Eocene:Cretaceous	**0.0247**	0.1236
Longest hastiseta length	Kruskal	0.1857
Setae/Body ratio	**Kruskal**	**2.85e-05**
Dunn test	uncorrected	corrected
Extant:Miocene	0.1195	0.3584
Extant:Eocene	0.1772	0.3544
**Extant:Cretaceous**	**2.36e-06**	**1.41e-05**
Miocene:Eocene	0.8595	0.8595
Miocene:Cretaceous	0.0837	0.3347
Eocene:Cretaceous	**0.0499**	0.2497
Hastisetae/body ratio	**Kruskal**	**0.0009**
Dunn test	uncorrected	corrected
Extant:Miocene	0.6825	0.6825
Extant:Eocene	0.2028	0.8111
**Extant:Cretaceous**	**0.0001**	**0.0007**
Miocene:Eocene	0.5549	1
Miocene:Cretaceous	0.0892	0.4457
Eocene:Cretaceous	0.2448	0.7345

**Megatominae**		
Body length	**Kruskal**	**0.0227**
Dunn test	uncorrected	corrected
Extant:Miocene	0.6047	1
Extant:Eocene	0.1503	0.4508
Extant:Cretaceous	**0.0108**	0.0645
Miocene:Eocene	0.1187	0.4746
Miocene:Cretaceous	**0.0328**	0.1642
Eocene:Cretaceous	0.8229	0.8229
Longest seta length	**Kruskal**	**0.0004**
Dunn test	uncorrected	corrected
Extant:Miocene	0.0756	0.3022
Extant:Eocene	0.4442	0.8883
**Extant:Cretaceous**	**5.07e-05**	**0.0003**
Miocene:Eocene	0.2861	0.8582
Miocene:Cretaceous	0.8074	0.8074
Eocene:Cretaceous	**0.043**	0.2148
Longest hastiseta length	Kruskal	0.1337
Setae/Body ratio	**Kruskal**	**4.36e-05**
Dunn test	uncorrected	corrected
Extant:Miocene	0.3647	0.7294
Extant:Eocene	0.2676	0.8027
**Extant:Cretaceous**	**3.71e-06**	**2.23e-05**
Miocene:Eocene	0.9575	0.9575
Miocene:Cretaceous	0.1461	0.5843
Eocene:Cretaceous	**0.0398**	0.1988
Hastisetae/body ratio	**Kruskal**	**0.0015**
Dunn test	uncorrected	corrected
Extant:Miocene	0.6657	0.6657
Extant:Eocene	0.1785	0.7139
**Extant:Cretaceous**	**0.0002**	**0.001**
Miocene:Eocene	0.5329	1
Miocene:Cretaceous	0.101	0.5052
Eocene:Cretaceous	0.2971	0.8912
