# Supplementary figures and images for: New Amber Fossils Indicate That Larvae of Dermestidae Had Longer Defensive Structures in the Past
Source: Insects. 2025 Jul 10;16(7):710. doi: 10.3390/insects16070710 (PMC12295104; doi:10.3390/insects16070710)

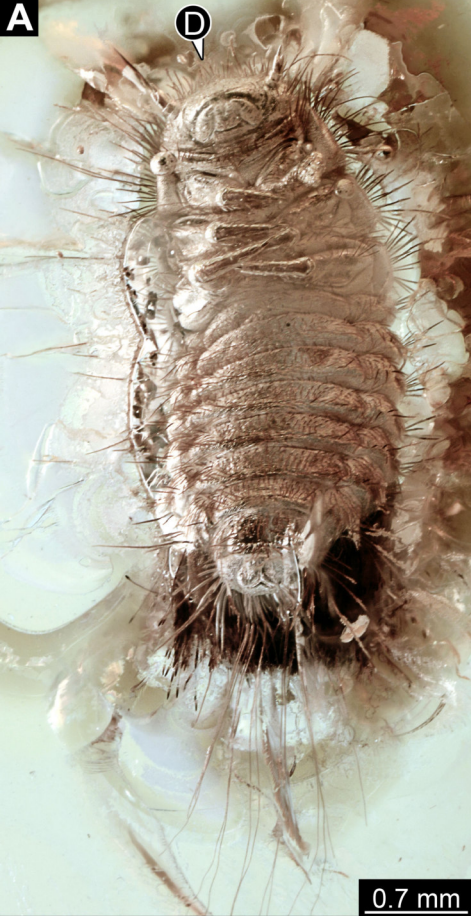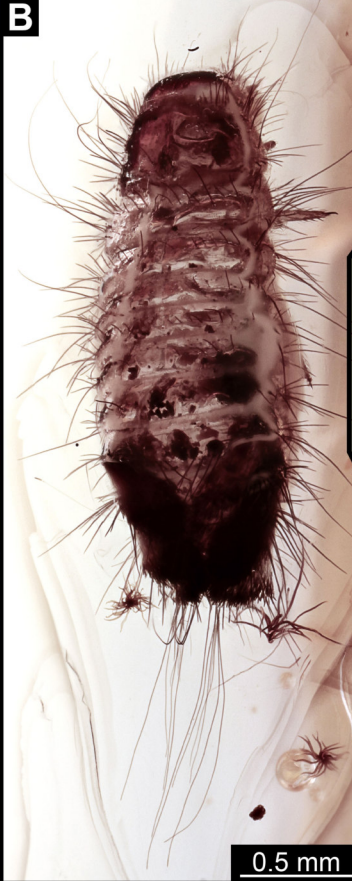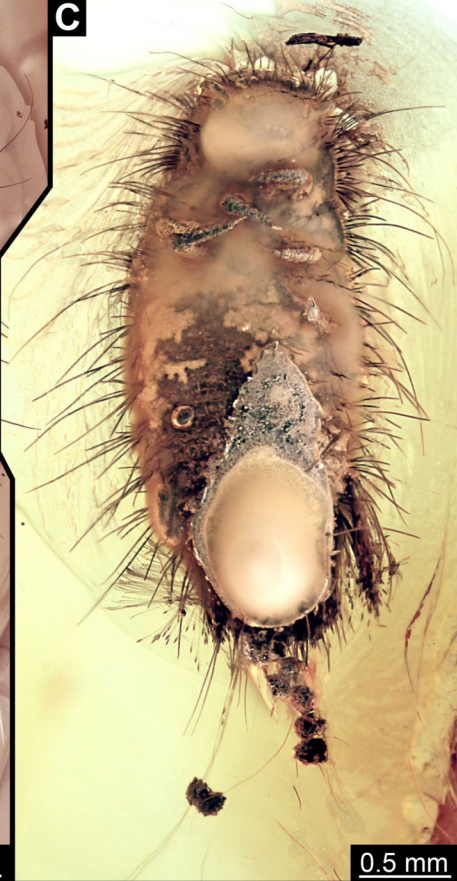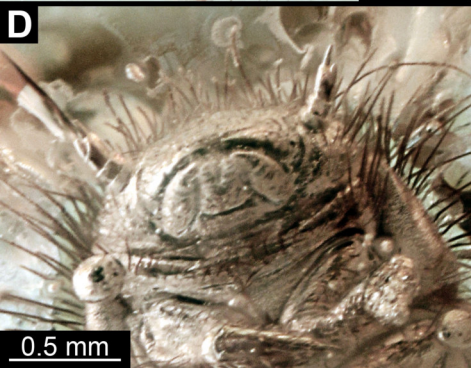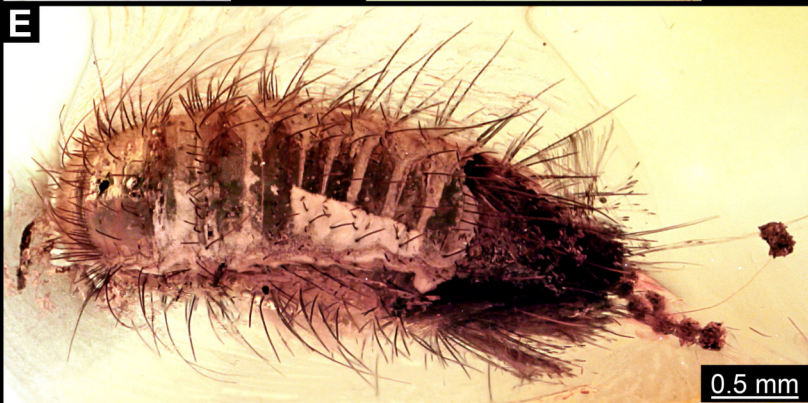

Supplement: Supplementary file 1 [file insects-16-00710-s001.zip › Supplementary Figure S1.pdf]

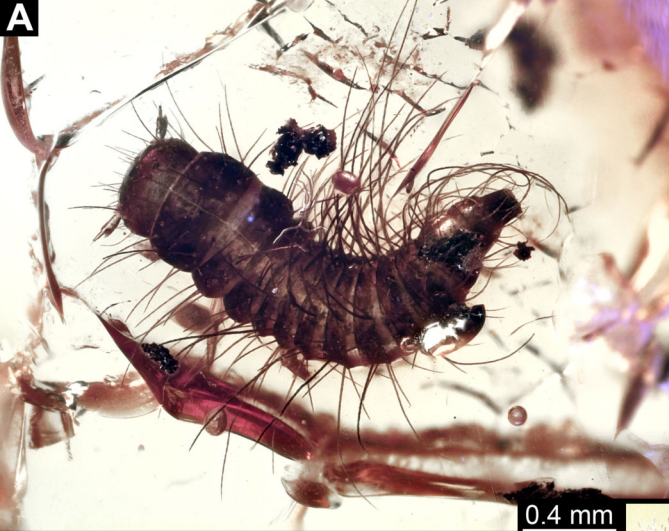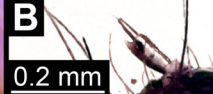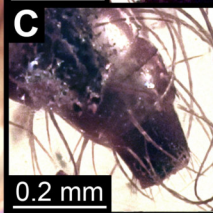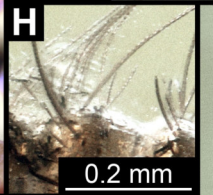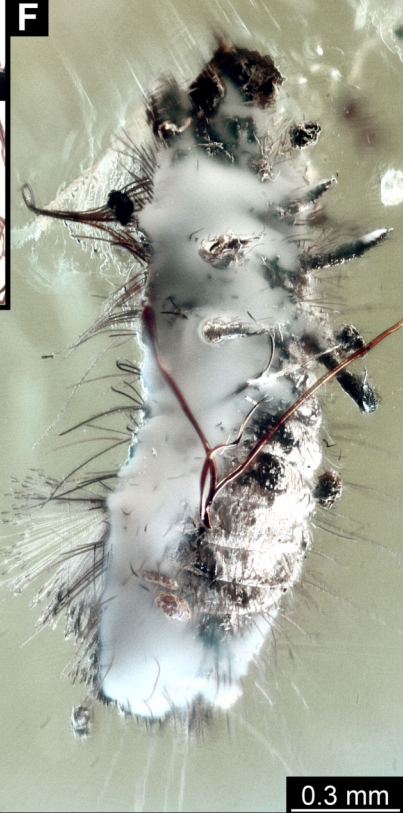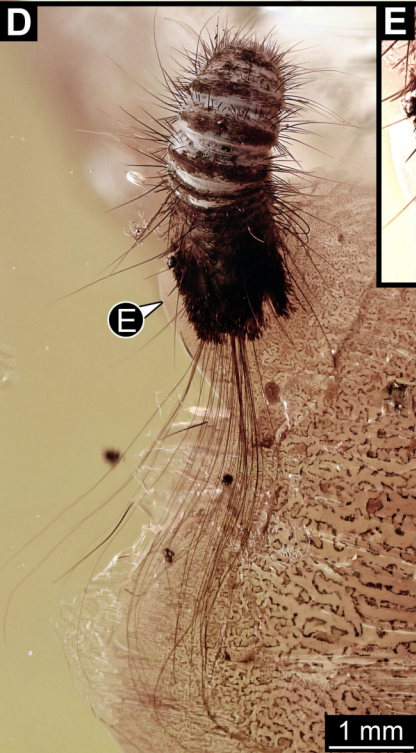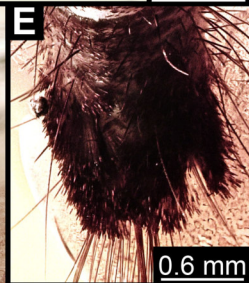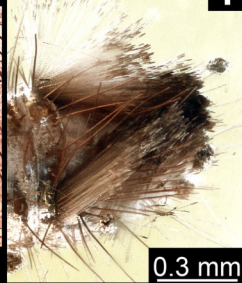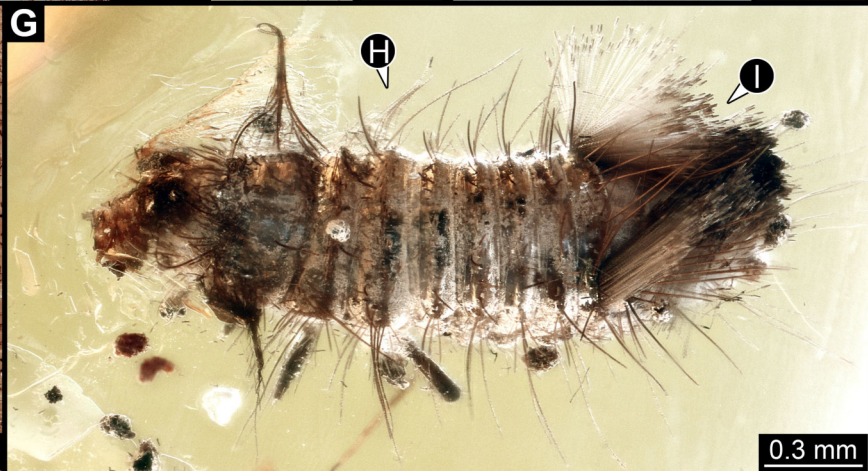

Supplement: Supplementary file 1 [file insects-16-00710-s001.zip › Supplementary Figure S2.pdf]

**A**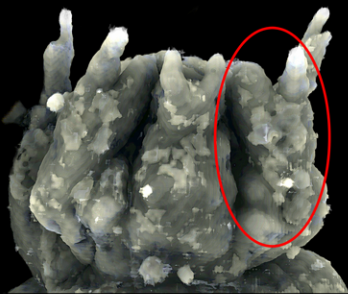**B**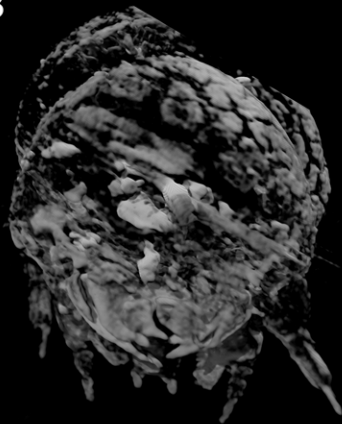**C**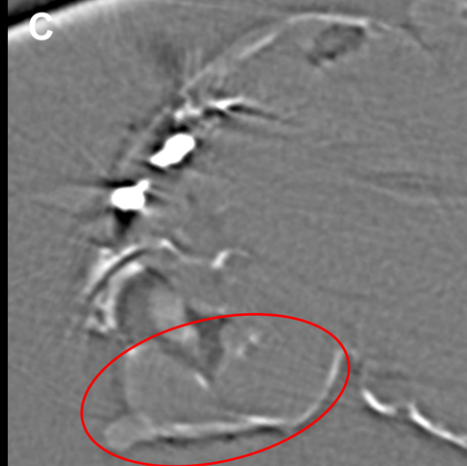**D**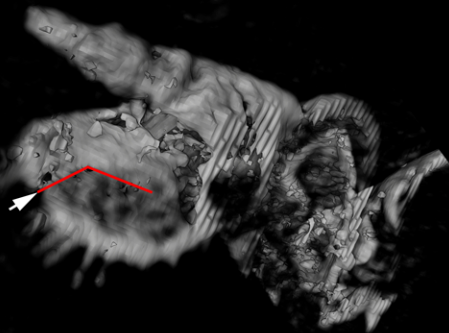**E**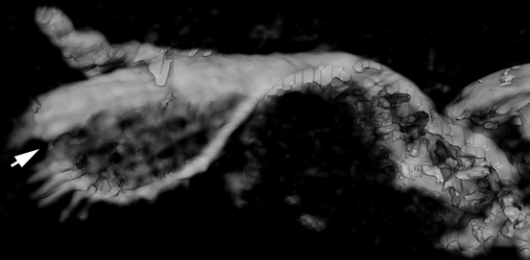**F**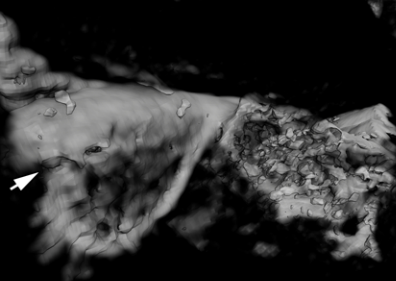**G**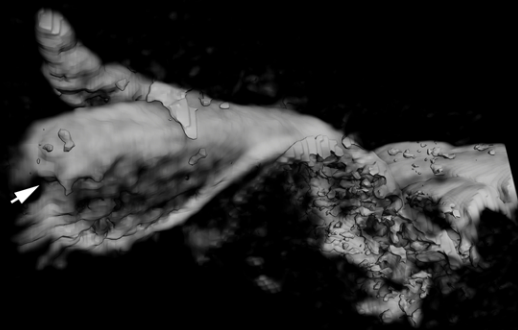**H**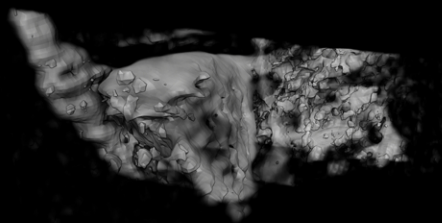

Supplement: Supplementary file 1 [file insects-16-00710-s001.zip › Supplementary Figure S3.pdf]
